# Genomic Instability of Osteosarcoma Cell Lines in Culture: Impact on the Prediction of Metastasis Relevant Genes

**DOI:** 10.1371/journal.pone.0125611

**Published:** 2015-05-19

**Authors:** Roman Muff, Prisni Rath, Ram Mohan Ram Kumar, Knut Husmann, Walter Born, Michael Baudis, Bruno Fuchs

**Affiliations:** 1 Laboratory for Orthopedic Research, Balgrist University Hospital, Zurich, Switzerland; 2 Institute of Molecular Life Sciences, University of Zurich, Zurich, Switzerland; University of Crete, GREECE

## Abstract

**Background:**

Osteosarcoma is a rare but highly malignant cancer of the bone. As a consequence, the number of established cell lines used for experimental *in vitro* and *in vivo* osteosarcoma research is limited and the value of these cell lines relies on their stability during culture. Here we investigated the stability in gene expression by microarray analysis and array genomic hybridization of three low metastatic cell lines and derivatives thereof with increased metastatic potential using cells of different passages.

**Principal Findings:**

The osteosarcoma cell lines showed altered gene expression during *in vitro* culture, and it was more pronounced in two metastatic cell lines compared to the respective parental cells. Chromosomal instability contributed in part to the altered gene expression in SAOS and LM5 cells with low and high metastatic potential. To identify metastasis-relevant genes in a background of passage-dependent altered gene expression, genes involved in "Pathways in cancer" that were consistently regulated under all passage comparisons were evaluated. Genes belonging to "Hedgehog signaling pathway" and "Wnt signaling pathway" were significantly up-regulated, and IHH, WNT10B and TCF7 were found up-regulated in all three metastatic compared to the parental cell lines.

**Conclusions:**

Considerable instability during culture in terms of gene expression and chromosomal aberrations was observed in osteosarcoma cell lines. The use of cells from different passages and a search for genes consistently regulated in early and late passages allows the analysis of metastasis-relevant genes despite the observed instability in gene expression in osteosarcoma cell lines during culture.

## Introduction

Osteosarcoma (OS) is a rare but highly malignant bone disease that affects predominantly children and adolescents. Patients with metastases still face a poor prognosis with a 5 year survival rate of less than 20% despite pre- and postoperative chemotherapy. Research in the field of OS is hampered by the low prevalence of the disease and by tumor cell heterogeneity. Moreover, OS is associated with chromosomal instability that appears to be caused by chromothripsis-like events that contribute to genomic heterogeneity in tumor cell populations [1–3]. Although the number of established OS cell lines is relatively low compared to other cancer entities, a few cell line systems are available for *in vitro* and *in vivo* research investigating mechanisms of OS progression [4]. These cell line systems consist of parental cell lines with a low metastatic potential, and derivatives thereof with increased metastatic activity *in vivo* [5–13]. The value of these systems for experimental OS research largely depends on the stability of the cell lines during culture. To our knowledge, the genomic stability in OS cell lines of these systems during serial passaging has so far not been investigated in detail. One previous study investigated the stability of a frequently used osteoblastic OS cell line (SAOS) during culture using functional assays and RT/PCR for an expression analysis of some selected genes [14]. The authors concluded that these cells are fairly stable, but that the expression of some selected genes differs considerably in cells derived from different passages. Another study concluded that osteoblastic OS cells derived from a primary tumor and a skip metastasis thereof remained stable for more than 100 passages, but no supporting data were included [13].

Malignant mesothelioma cells showed increasing chromosomal abnormalities during culture associated with deregulated gene expression assessed by array comparative genomic hybridization (aCGH) and microarray gene expression analysis [15]. Using a proteomic approach, instability in protein expression during culture was also described in lung adenocarcinoma cells [16]. A study using microarray gene expression analysis in oral cancer cell lines showed that a considerable number of genes is differentially expressed during culture despite the fact that serial passaging had no significant effect on global gene expression of cancer related genes [17]. Chromosomal instability together with *in vitro* evidence for an increased transformed phenotype was observed during culture of spontaneously immortalized but non-tumorigenic keratinocytes and in lung epithelial cells, in a spontaneously immortalized non-tumorigenic breast epithelial cell line and in ovarian cancer cells [18–21]. However, tumorigenicity was reduced during culture of melanoma cells [22].

Stimulated by the lack of information on the stability of OS cells, we investigated in the present study global changes in gene expression during culture of frequently used human (SAOS/LM5 and HOS/143B [5,7]) and mouse (Dunn/LM8 [9]) OS cell line systems. The results showed limited stability of gene expression in the parental low metastatic cell lines (SAOS, HOS, Dunn), and remarkably increased instability during culture of the metastatic derivatives of the two osteoblastic cell lines (LM5 and LM8), the most common OS subtype. Passage related changes in gene expression may influence the data analysis of microarray studies addressing the search for metastasis relevant genes in OS. In an attempt, described herein, to search for passage-independent metastasis relevant genes, we were able to identify several genes that are up-regulated in highly compared to low metastatic cell lines in the investigated systems and belong to groups of genes involved in the regulation of hedgehog and WNT signaling pathways.

## Materials and Methods

### Cell lines and culture

Human SAOS (HTB-85), HOS (CRL-1543) and 143B (CRL-8303) cells were obtained from ATCC (Rockville, MD). Human LM5 cells, derived from parental SAOS cells [5], were kindly provided by E.S. Kleinerman (M.D. Anderson Cancer Center, Houston, TX). The mouse Dunn cells, originally established by T. B. Dunn [23], and the LM8 cells, derived from parental Dunn cells [9] were provided by T. Ueda (Osaka University Graduate School of Medicine, Osaka, Japan). Animal experiments for the generation of LM5 and LM8 cells were approved by the local ethic committees (personal communication from E. Kleinerman and T. Ueda). Cells were cultured in DMEM (4.5 g/l glucose)/F12 (1:1) medium supplemented with 10% heat-inactivated fetal calf serum in a humidified atmosphere of 5% CO_2_. Cells were split at ratios and for passage numbers given in Table A in S1 File when they reached near confluence to ensure that proliferation was maintained throughout the culture process. Authentication of the human cell lines was performed at the early and late passages by multiplex PCR (Microsynth, Balgach, Switzerland) using the PowerPlex16HS system (Promega, Madison, WI) and verified by comparison with the database at the German Collection of Microorganisms and Cell Cultures (DSMZ, Braunschweig, Germany). The cells were free of mycoplasma and not contaminated by other human cell lines. The mouse cell lines were also mycoplasma free and not contaminated by human cell lines.

Aliquots of early passage cells were frozen. Late passage cells, derived from early passage cells, as described above, were also frozen after they reached the required doublings. For RNA and DNA extraction and microarray and aCGH analysis, respectively, early and late passage cells were thawed and cultured for additional three passages under identical culture conditions.

### RNA extraction and Agilent GeneChip processing

Sub-confluent cells were detached with trypsin/EDTA, centrifuged and cell pellets immediately frozen in liquid nitrogen and stored at -80°C until RNA extraction. Total RNA was isolated from frozen cell pellets of individual cell lines (three samples per condition) with TriReagent (Sigma-Aldrich, St. Louis, MO) as described [24]. The RNA was quantified by measuring the absorption at 260 and 280 nm in a UV-spectrophotometer. The integrity of the RNA was assessed by standard agarose gel-electrophoresis and with a Bioanalyzer 2100 (Agilent Technologies, Palo Alto, CA). Complementary RNA preparation and array hybridization was performed by the Functional Genomics Center (Zurich, Switzerland). Microarray data for human cell lines were generated with Agilent SurePrint Human Gene Expression 8x60k microarray kit (chip ID: 028004) containing 42,545 probe sets that match to 21,339 genes identifiable by the Kyoto Encyclopedia of Genes and Genomes (KEGG) number (Agilent, Santa Clara, CA). For the mouse cell lines, Agilent Mouse GE 4x44k v2 microarray kit (chip ID: 026655) was used containing 39,485 probe sets that match to 23,074 genes identified by KEGG number. The chromosomal distribution of genes is presented in Table B in S1 File.

### cDNA synthesis and real-time PCR analysis

Total RNA (1 μg of the RNA used for the microarray analysis) was reverse-transcribed to cDNA with a high capacity RNA-to-cDNA kit (Applied Biosystems, Foster City, CA) in a final volume of 20 μl according to the protocol provided by the manufacturer. RNA of three independent extracts from the individual cell lines was reverse-transcribed. Real-time PCR was carried out in a StepOne Plus Real-Time PCR system (Applied Biosystems) in 96 well plates. Primers (Table C in S1 File) were designed for amplification of cDNA sequences derived from selected genes using NCBI Primer blast (http://www.ncbi.nlm.nih.gov/tools/primer-blast/) software. PCRs from individual RT reactions were carried out in triplicates. cDNA equivalent to 50 ng of RNA and appropriate primers were added to Power SYBR Green PCR master mix (Applied Biosystems) and the samples were pre-incubated at 50°C for 2 min and at 95°C for 10 min and then subjected to 40 cycles of incubation at 95°C for 15s and at 60°C for 1 min. The threshold for Ct values was set to 0.325. To verify the amplification of a single product in any of the PCR reactions, a melting curve was generated and analysed after every run. Relative expression levels were calculated by the comparative cycle threshold (ΔΔCT) method and were normalized to GAPDH expression.

### DNA extraction and Affymetrix CytoScan HD array

RNA-free DNA from the individual OS cell lines was extracted using the Gentra Puregene kit (Qiagen, Hilden, Germany) according to the protocol recommended by the supplier. DNA concentration and purity was determined with a NanoDrop 1000 spectrophotometer (Thermo Scientific, Wohlen, Switzerland). Since non-degraded DNA was needed, the size of the double-stranded genomic DNA was assessed on a 1% agarose gel. DNA was concentrated and resuspended in nuclease-free water, and the concentration was measured with a NanoDrop 1000 spectrophotometer. Microarray CGH was performed with the CytoScan HD Array Kit (Affymetrix, Santa Clara, CA) according to the instructions of the manufacturer at the Laboratory for Oncology Diagnostics (Kinderspital Zurich, Switzerland). The files obtained from the scanner were analyzed with softwares from different companies: the Affymetrix Chromosome Analysis Suite (ChAS) 2.0.0.195 and the BioDiscovery Nexus Copy Number 7.0. Copy number variants (CNV) according to the Database of Genomic Variants (http://dgv.tcag.ca/dgv/app/home), DNA copy number alterations smaller than 100 Kb or 50 adjacent probes and loss of heterozygosity (LOH) smaller than 3 Mb were not considered if not known as recurrent aberrations for any neoplasia.

### ArrayMap and KEGG pathway analysis

Called copy number aberrations were combined with a published reference dataset from an SAOS genome array (GSM170249; [25], all (re)mapped to Human Genome version 19. Changes in relative copy numbers between early and late passage SAOS and LM5 cells were mapped and evaluated with respect to their overlap with the genome positions of top scoring genes showing the corresponding expression change (i.e. genes with increased expression vs. regions with relative copy number gains), using custom software implementations. Ratios of corresponding gains and losses and same directional expression changes were calculated compared to the expected values of a random genomic distribution of genes with altered expression values.

Chromosomal and cytoband localization of differentially expressed genes (>2-fold; p<0.05) and KEGG pathway analysis was performed using ''The **D**atabase for **A**nnotation, **V**isualization and **I**ntegrated **D**iscovery'' (**DAVID**) v6.7 (http://david.abcc.ncifcrf.gov/) [26] by entering the Entrez gene ID lists of regulated genes. To compare human and mouse arrays the IDs were converted using MADGENE (http://cardioserve.nantes.inserm.fr/mad/madgene/).

Microarray and aCGH data were deposited in GEO under the accession numbers GSE66673, GSE66674 and GSE67125, respectively.

### Statistical analysis

P and fdr (false discovery rate) values in microarray analysis were obtained after two group analysis of triplicate samples using *t* test and Benjamini-Hochberg fdr according to with Bioconductor software package limma (http://www.bioconductor.org/packages/release/bioc/html/limma.html) [27]. P values for the enrichment of regulated genes were obtained by a modified Fisher’s exact test (EASE score) using DAVID (http://david.abcc.ncifcrf.gov/) [26].

## Results

### Number of genes undergoing differential expression during serial passaging

**Fig 1:**
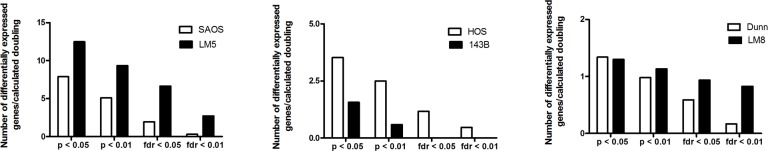
Number of differentially expressed genes after serial passaging. The number of differentially expressed genes at different significance levels was normalized to calculated doublings in order to compare the three cell line systems. Note the different y-axis scales for each cell line system. fdr; false discovery rate.

The parental human SAOS, HOS and mouse Dunn cells with low metastatic potential were cultured to achieve >150 doublings, respectively (Table A in S1 File). A total of 1297, 1105 and 626 genes were differentially expressed (>2-fold; p<0.05) in late versus early passages in SAOS, HOS and Dunn cells, respectively. The number of differentially expressed genes per doubling was 7.9, 3.5 and 1.3 for SAOS, HOS and Dunn cells, respectively (Fig 1). Therefore, considerable differences in the stability of gene expression during serial passaging of parental cell lines are observed. The *in vivo* selected sub-lines LM5 and LM8 with increased metastatic potential, derived from parental SAOS and Dunn cells, respectively, were cultured to achieve similar doublings as compared to parental cell lines. In LM5 and LM8 cells 12.5 and 1.3 genes were differentially expressed per doubling. LM5 cells, therefore, seem to be less stable than parental SAOS cells and this became even more pronounced at higher stringency levels (Fig 1). A similar trend was observed for LM8 cells, which also presented with more differentially expressed genes than the Dunn cells at higher stringency levels. The metastatic 143B cells, derived from HOS cells by Ki-ras transformation, presented 430 differentially expressed genes after 275 doublings, corresponding to 1.7 differentially expressed genes per doubling. In this cell line system, the metastatic subline was more stable than the parental cell line. Taken together, the results imply that OS cells display limited stability in gene expression during culture and that the instability is more pronounced in the *in vivo* selected metastatic derivatives than in the parental cell lines.

In human SAOS and LM5 cells, 810 (3.80%) and 1035 (4.85%) genes, respectively, were up-regulated (>2-fold; p<0.05) with increasing passage number and 487 (2.28%) and 912 (4.27%) genes were down-regulated. 201 (0.94%) up-regulated and 94 (0.44%) down-regulated genes were found to be the same in the two cell lines; these numbers were 5.2- and 4.5-times higher than statistically expected (0.18% and 0.097% for up- and down-regulated genes, respectively). Similarly, in mouse Dunn and LM8 cells, 313 (1.36%) and 327 (1.42%) genes were up-regulated and 313 (1.36%) and 338 (1.47%) genes were down-regulated, respectively, after serial passaging. The two lines had 33 (0.14%) up- and 66 (0.29%) down-regulated genes in common and these numbers were 7.4- and 14.5-times higher than statistically expected (0.019% and 0.02% for up- and down-regulated genes, respectively). In contrast, in human HOS and 143B cells, only 24 common up-regulated and 3 common down-regulated genes were found and these numbers were only 2.1- and 1.7-times higher than statistically expected. This indicates a significant common change in gene expression patterns in the parental low metastatic cell lines and the metastatic derivatives during serial passaging in the human SAOS/LM5 and the mouse Dunn/LM8 cell line systems, but not in the human HOS/143B cell line system in which the changes in gene expression patterns were largely different.

To compare the number of differentially expressed genes after serial passaging with that of the comparison of the metastatic versus the parental cell lines, we pooled the data sets of early and late passages of each parental and metastatic cell line before the comparison between low and highly metastatic cell lines was performed. In the most unstable SAOS/LM5 cell system, the sum (3244) of differentially expressed genes after serial passaging of SAOS and LM5 cells was in the same range as after the comparison between low and highly metastatic cell lines (2578). In the other two cell line systems, the comparison between low and highly metastatic cell lines yielded higher numbers of differentially expressed genes than the sum of differentially expressed genes in respective cell lines after serial passaging (Table D in S1 File). Altered gene expression after serial passaging may nevertheless affect the analysis of metastasis-relevant genes in all these cell systems.

### Chromosomal localization of genes differentially expressed after serial *in vitro* passaging

After serial passaging, significant (p<0.01) clustering of up- or down-regulated genes on distinct chromosomes was observed in all cell lines except in 143B cells, the most stable cell line during *in vitro* culture (Table D in S1 File). In SAOS and LM5 cells, such clusters of differentially regulated genes were found on four and seven chromosomes, respectively. Up-regulated genes in SAOS (64 genes) and LM5 (99 genes) cells were found enriched on chromosome 12. Based on the fact that 22 of the up-regulated genes on chromosome 12 were found to be identical in the two cell lines and only two were expected by chance, corresponding chromosomal loci appear to be hot spots for changes in gene expression during serial passaging of SAOS and LM5 cells. The same applies to 22 genes (only 4 expected by chance) located on chromosome 1. In SAOS cells, 28 up-regulated genes clustered in the central to distal region of the long arm of chromosome 1 from 1q41-1q42.13, and 21 genes clustered in the distal region 12p13.31-12p13.33. In LM5 cells, up-regulated gene expression clustered in distal parts in 4p16.1-4p16.3 (41 genes), 12p13.31–12p13.33 (21 genes) and 16p13.3 (18 genes), whereas down-regulated genes clustered in the central part of 10p12.1-10p12.31 (17 genes) and in adjacent 10p13 (10 genes). Of the 22 genes on chromosome 12 found up-regulated in both, the SAOS and LM5 cells, 11 genes map to the region 12p13.31-12p13.33. Taken together, all findings support the notion that genes, which undergo differential expression during serial passaging of SAOS and LM5 cells are not randomly distributed in the genome, but are located in clusters on distinct chromosomes or even in cytobands. Even though more chromosomes and regions thereof are affected in the metastatic LM5 cells than in parental SAOS cells, some of the hot spots of differentially expressed genes identified in the two cell lines were found to be identical.

Serial passaging of HOS cells revealed preferential down-regulation of genes on chromosomes 17 (28 genes) and 19 (50 genes) whereas in 143B cells no enrichment on distinct chromosomes was recognized. Clustering of 11 down-regulated genes in the central region 5q31 and of 12 up-regulated genes in distal region 19p13.3 was noted in HOS cells. In 143B, no cytobands were affected to any great extent.

In serially passaged mouse Dunn and LM8 cells, differentially regulated genes were located on three and one chromosomes, respectively. In Dunn cells, up-regulated genes were mainly found on chromosomes 14 (24 genes) and 18 (20 genes), and down-regulated genes on chromosome 9 (24 genes). In LM8 cells preferential up-regulation was observed only on chromosome X (46 genes). Clustering was low with three and one affected cytobands in Dunn and LM8 cells, respectively, containing only 9 to 11 genes. Taken together, chromosomal events during culture, which remain to be defined, appear to contribute to differential gene expression predominantly in the SAOS/LM5 cell system and to a lesser extent in the other two systems.

During selection for metastatic activity of OS cell lines in the individual systems, changes in gene expression occurred on more chromosomes than during serial passaging (Table D in S1 File). In the SAOS/LM5 cell system, 11 of the 22 autosomes and the X chromosome showed significant enrichment of up- and down-regulated gene expression. In the HOS/143B cell system 9 autosomes were affected, and in the Dunn/LM8 cell system 6 of the 19 autosomes exhibited significant enrichment of differentially expressed genes. Taken together, chromosomal instability appears to contribute to altered gene expression during culture and to a greater extent during selection for increased metastatic activity of OS cell lines.

### Analysis of chromosomal aberrations by aCGH in SAOS and LM5 cells subjected to serial passaging or selection for increased metastatic activity

**Fig 2:**
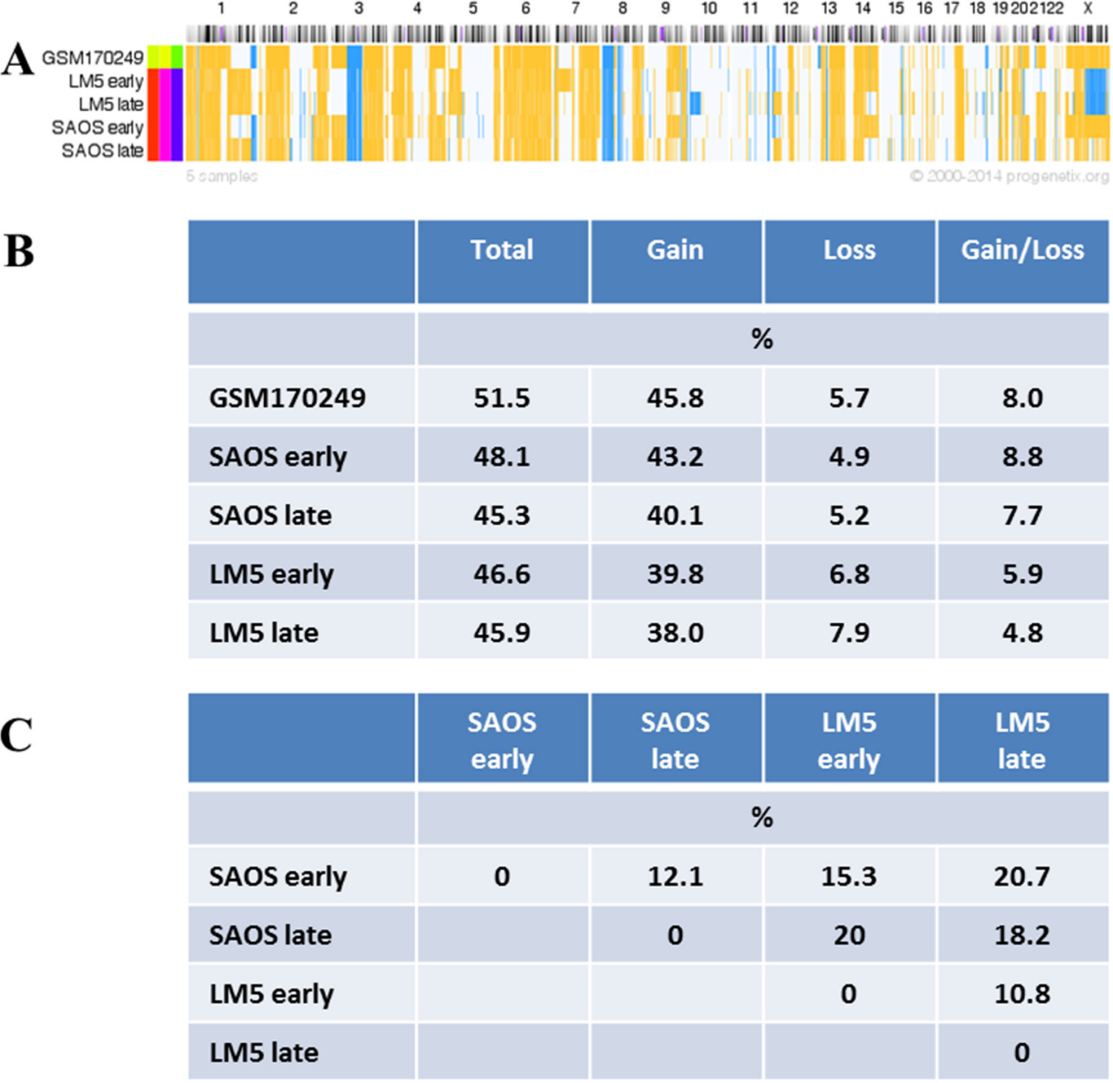
Copy number (CN) aberrations in the SAOS/LM5 cell system. (A) CN gains (yellow) and losses (blue) compared to normal diploid human genome were analyzed in SAOS and LM5 cells of early and late passages and compared to published data of SAOS cells (GSM170249; [25]) using arrayMap as described in *Methods*. (B) Statistics of CN gains and losses compared to normal human diploid genome. (C) Total CN differences between early and late passages of SAOS and LM5 cells.

OS display on average a high rate of genomic imbalances (25% genome fraction vs. ~14% in all cancer entities; data based on [28]). In the present study, an analysis of copy number (CN) gains and losses was carried out by aCGH analysis in SAOS and LM5 cells at early and late passages (Fig 2A). The analysis of published chromosomal aberrations in SAOS cells (GSM170249; [25]) revealed differences in CN of 52% of the genes of the normal human diploid genome, consisting of 46% gains and 6% losses in CN (Fig 2B). Our population of early passage SAOS cells showed 9% CN differences compared to GSM170249, with a total aberrant fraction of 48% consisting of 43% gains and 5% losses in CN compared to the diploid genome.

During serial passaging of SAOS and LM5 cells, changes in CN between early and late passages involved 12.1% (4.4% gain and 7.7% loss) and 10.8% (3.9% gain and 6.9% loss) of the genes of the entire genome, respectively (Fig 2C). The largest difference (20.7%) of total CN variation was observed when early passage SAOS cells were compared to late passage LM5 cells.

“Macro”-aberrations, defined as loss or gain of at least one cytoband, during serial passaging of SAOS and LM5 cells, are summarized in Table E in S1 File. Seventeen macro-aberrations were identified in both SAOS (5 gains and 12 losses) and LM5 cells (9 gains and 8 losses). Interestingly, 6 out of 17 chromosome regions with CN changes during culture were the same in the two cell lines. They included 3q (loss), 9q (loss), 12p (gain) and 14q (loss). A comparison of the list of the up- and down-regulated genes obtained by microarray analysis with the regions of dynamic CN status found by aCGH analysis revealed a 2–2.2 fold ratio of observed over expected total coincidence (Table F in S1 File). A 3-fold most prominent coincidence was found for up-regulated genes in late versus early LM5 cells. This indicates that a substantial fraction of observed expression differences following serial passaging results from changes in CN of the affected genes. “Hotspots" of coincidence in changes of gene CN and expression were up-regulated genes in 1q and 12p in late passage SAOS cells and up-regulated genes in 4p, 12p and 21q in late passage LM5 cells (Fig A in S1 File). Coincidence of down-regulated genes and CN loss was observed in late passage LM5 cells in 9q and 10p. These results largely confirm the above described significant clustering of differentially expressed genes in distinct chromosomes and cytobands (Tables D and E in S1 File).

Macro-aberrations, defined as losses or gains in CN in LM5 compared to SAOS cells, which occurred independent of passage number during selection for increased metastatic activity are summarized in Table G in S1 File. Seventeen regions (8 gains and 9 losses) with macro-aberrations were identified on 10 chromosomes. The largest changes in CN observed were losses in 3p, 4q, 8q, 20q and Xq and gains in 1q, 5p, 15q and 20p. Thus, considerable changes in CN in distinct regions of individual chromosomes occurred also during selection for increased metastatic activity of LM5 compared to SAOS cells, but in locations different from those that showed CN changes during serial passaging of the individual cell lines.

### Genes deregulated after serial passaging

Pathway analysis of differentially expressed genes in early and late passage cells revealed a significant enrichment (p<0.05) of genes involved in the "Focal adhesion" pathway in all cell lines investigated except in 143B cells (Table 1). Genes of all other pathways listed in Table 1 were only significantly enriched in three out of the six cell lines investigated. The analysis also showed that genes, which were differentially regulated in both the SAOS and LM5 cell lines at early and late passages belonged to pathways involved in "Focal adhesion", "Cytokine-cytokine receptor interaction" and "PPAR signaling pathway" (not listed in Table 1). Similarly, in the Dunn/LM8 cell line system, genes belonging to the three pathways "Focal adhesion", "ECM-receptor interaction" and "Pathways in cancer" were differentially regulated in both cell lines upon serial passaging. In the HOS/143B cell systems, on the other hand, only genes involved in "Neuroactive ligand-receptor interaction" were differentially regulated in both cell lines.

**Table 1:** Number of differentially regulated genes enriched in KEGG pathways after serial passaging of indicated cell lines.

KEGG pathways	SAOS	LM5	HOS	143B	Dunn	LM8
Focal adhesion	**22** (13/9)	**33** (**20**/13)	**20** (12/8)		**16** (**9**/7)	**20** (3/**17**)
Cytokine-cytokine receptor interaction	**30** (18/**12**)	**37** (15/**22**)	**25** (15/10)			
ECM-receptor interaction		**19** (9/**10**)			**9** (3/**6**)	**12** (1/**11**)
Neuroactive ligand-receptor interaction	**26** (17/9)		**24** (**17**/7)	**9** (5/4)		
TGF-beta signaling pathway		**16** (**11**/5)	**21** (**9**/**12**)		**8** (4/4)	
Pathways in cancer			**27** (**20**/7)		**21** (**13**/8)	**26** (**12**/**14**)

Pathways are listed that are significantly (**p<0.05**) enriched in at least three cell lines. The numbers in brackets indicate up-/down-regulated genes (>2-fold; p<0.05). Comparison is performed from pooled data sets from early and late passages.

In SAOS and LM5 cells 13 and 20 genes, respectively, involved in "Focal adhesion" were up-regulated in late versus early passage, and four genes (CCND1, ITGA4, PARVG and MYL10) were up-regulated in both cell lines. This was confirmed by real-time PCR analysis (Fig B in S1 File), in which CCND1, ITGA4 and PARVG (3 out of 4) genes also showed a > 2-fold increase in expression in late versus early passage. No gene involved in "Focal adhesion" was found down-regulated in both the SAOS and LM5 cell lines. Thirty and 37 genes belonging to "Cytokine-cytokine receptor interaction" were enriched in SAOS and LM5 cells, respectively. The two cell lines had 6 down-regulated (IL15, IL15RA, IL20RB, BMPR1B, EDA and INHBE) and five up-regulated genes (TNFRSF8, TNSF10, CNTFR, NGFR and CXCL14) in common. In the "PPAR signaling pathway" 11 and 13 genes were enriched in SAOS and LM5 cells, respectively. FABP6 was up-regulated and PCK2 and SCD5 were down-regulated in both cell lines.

In the Dunn and LM8 cell lines, 7 and 17 genes, respectively, of the "Focal adhesion" pathway were down-regulated in late versus early passage and three genes (COL4A1, COL4A2 and ITGA11) were down-regulated in both cell lines. These genes are also involved in "ECM-receptor interaction" and are the only genes in this pathway, which are regulated in both Dunn and LM8 cells. None of the up-regulated genes involved in "Focal adhesion" and "ECM-receptor interaction" was found altered in both Dunn and LM8 cells. Interestingly, in the Dunn/LM8 cell line system also genes involved in "Pathways in cancer" were significantly deregulated during serial passaging. No up-regulated genes, but four down-regulated genes (COL4A1, COL4A2, EPAS1 and FGF15) of the "Pathways in cancer" were found in both cell lines.

In the HOS/143B cell line system, only genes involved in "Neuroactive ligand-receptor interaction" were significantly deregulated, but with minimal commonly affected genes. But in HOS cells, like in the Dunn/LM8 cell line system, also genes of the "Pathways in cancer" were deregulated during serial passaging.

Taken together, the results of the pathway analysis imply deregulation of genes involved in "Focal adhesion" during serial passaging in all three cell line systems. A partial common shift in gene expression of genes belonging to other pathways was also observed in the SAOS/LM5 and the Dunn/LM8, but not the HOS/143B cell systems. Moreover, some genes deregulated in during serial passaging might also play a role in cancer.

### Genes deregulated after selection for increased metastatic activity

In order to analyze genes that increase the metastatic activity of LM5, 143B and LM8 cells, independent of the observed *in vitro* instability, two approaches were followed. First, the expression data sets of early and late passaged cells were pooled before the comparison of the data obtained for the metastatic and parental cell lines was performed for each of the three cell line systems. Second, the comparison between low and highly metastatic cell lines was performed for each of the four individual combinations of passage data sets (metastatic early/parental early; metastatic late/parental early; metastatic early/parental late; metastatic late/parental late).

Pathway analysis of the pooled data sets revealed significant enrichment of differentially regulated (p<0.05) genes belonging to "Pathways in cancer", "Basal cell carcinoma", "Focal Adhesion", "ECM-receptor interaction" and "Wnt signaling pathway" in all three cell line systems (Table 2). 56, 95 and 59 genes belonging to "Pathways in cancer" were differentially regulated in the SAOS/LM5, HOS/143B and Dunn/LM8 cell systems, respectively. As genes belonging to some of the pathways were also significantly deregulated during serial passaging of parental and/or metastatic cell lines, we searched for genes belonging to "Pathways in cancer" that were consistently regulated (>2-fold; p<0.05) in all individual combinations of early and late passage data sets of metastatic and parental cell lines. Compared to the pooled analysis, the number of differentially regulated genes was now reduced to 14 (25%), 58 (61%) and 29 (49%) genes in the SAOS/LM5, HOS/143B and Dunn/LM8 cell systems, respectively. The herewith identified potential metastasis-relevant genes for each cell line system together with the fold regulation are listed in Table H in S1 File.

**Table 2:** Number of differentially regulated genes enriched in KEGG pathways in high and low metastatic cell lines.

KEGG pathways	LM5/SAOS	143B/HOS	LM8/Dunn
Pathways in cancer	**56** (**29**/27)	**95** (**49**/**46**)	**59** (**32**/**27**)
Basal cell carcinoma	**18** (**11**/7)	**21** (**13**/8)	**13** (**11**/2)
Focal adhesion	**36** (16/20)	**67** (24/**43**)	**40** (14/**26**)
ECM-receptor interaction	**23** (8/**15**)	**37** (12/**25**)	**16** (4/**12**)
Wnt signaling pathway	**29** (13/**17**)	**41** (**25**/16)	**31** (**25**/6)
Bladder cancer		**14** (**9**/5)	**13** (**8**/5)
Colorectal cancer		**29** (**19**/10)	**18** (**14**/4)
Small cell lung cancer		**31** (**15**/**16**)	**17** (7/**10**)
ErbB signaling pathway		**24** (13/11)	**16** (10/6)
MAPK signaling pathway		**76** (**36**/**40**)	**40** (22/**18**)
Hedgehog signaling pathway	**16** (**11**/5)	**19** (**12**/7)	
Calcium signaling pathway	**34** (12/**21**)	**54** (22/**32**)	
Gap junction		**27** (14/14)	**17** (**13**/4)
Adherens junction		**22** (11/11)	**15** (10/5)

Pathways are listed that are significantly (**p<0.05**) enriched in at least two cell line systems. The numbers in brackets indicate up-/down-regulated genes (>2-fold; p<0.05). Comparison is performed from pooled data sets from early and late passages.

**Fig 3:**
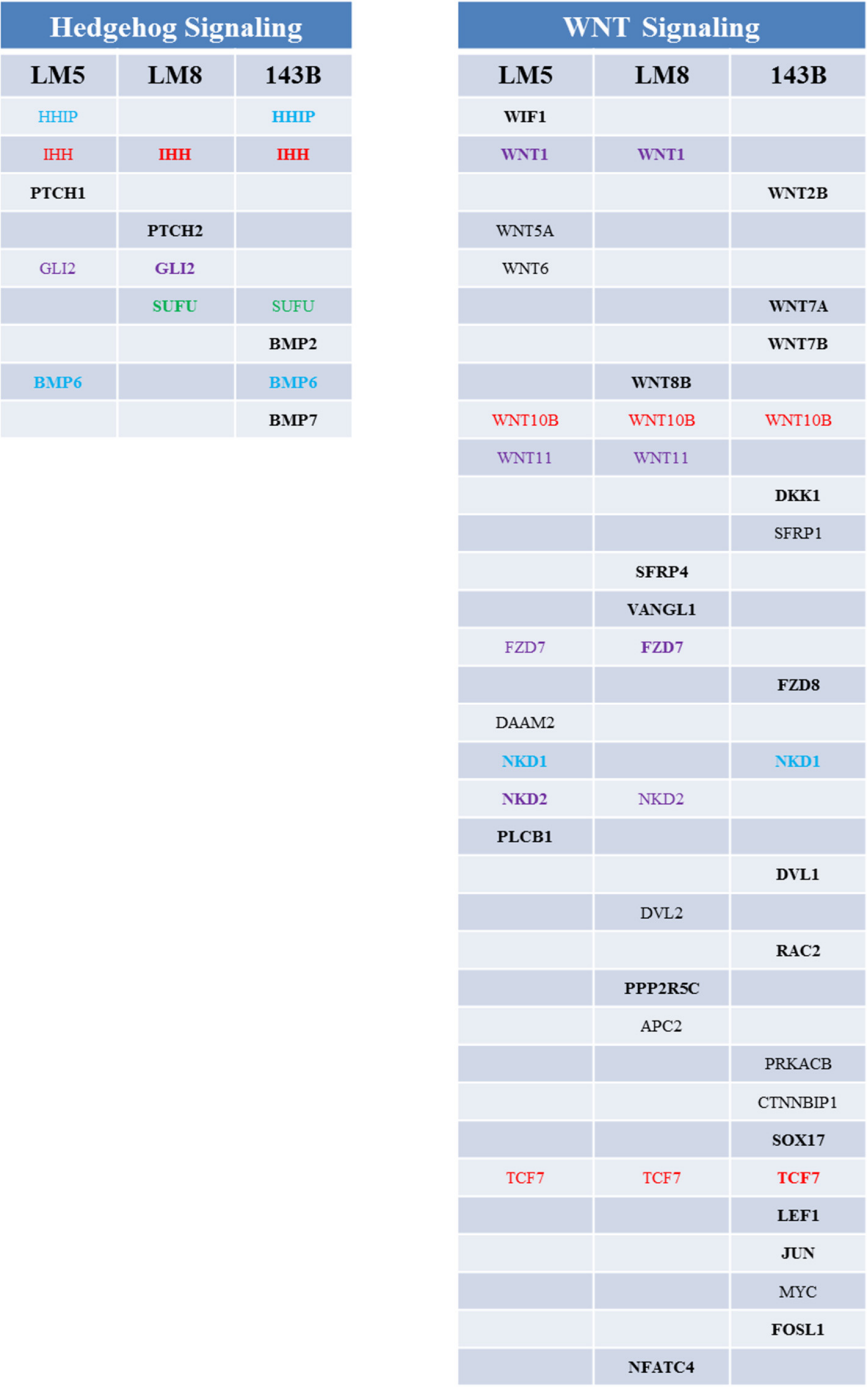
Up-regulated genes involved in hedgehog and WNT signaling in metastatic cell lines. Indicated genes are up-regulated (>2-fold; p<0.05) in at least three passage comparisons. Genes up-regulated in four passage comparisons are shown in bold. Genes marked in red are up-regulated in all three metastatic cell lines. Genes marked in purple, blue and green are shared by LM5 and LM8, LM5 and143B and LM8 and 143B, respectively.

Among the signaling pathways the "Wnt signaling pathway" and the "Hedgehog signaling pathway" also showed significant enrichment in the pathway analysis of the pooled data sets (Table 2) and both pathways are known to play an important role in bone development [29]. Genes involved in these pathways were also deregulated after serial passaging of parental and/or metastatic cell lines (Table I in S1 File), with the SAOS/LM5 cell system being the most affected. Therefore, we performed again individual metastatic versus parental comparisons of early and late data sets that revealed significant enrichment of up-regulated genes that belong to "Hedgehog signaling pathway" (3 of 4 comparisons) in all three cell line systems (Table J in S1 File). Up-regulation of the "WNT signaling pathway" was observed in LM8 (3 comparisons) and 143B cells (4 comparisons). The combined lists of up-regulated genes (>2-fold; p<0.05) that are regulated under at least three comparisons together with the fold regulation is shown in Table K in S1 File. In the SAOS/LM5, HOS/143B and Dunn/LM8 cell systems 17, 24 and 17 genes, respectively, belonging to "Wnt signaling pathway" and/or "Hedgehog signaling pathway" were up-regulated after selection for increased metastatic potential. In the SAOS/LM5, HOS/143B and Dunn/LM8 cell systems 7 (41%), 18 (75%) and 10 (59%) genes, respectively, were significantly up-regulated in all four comparisons. This is consistent with the observation that in the SAOS/LM5 cell system, 11 of the 17 listed genes (65%) were also deregulated after serial passaging, whereas in the HOS/143B and Dunn/LM8 cell system only 21% and 18%, respectively, of the genes were differentially expressed upon serial passaging. IHH, WNT10B and TCF7 were up-regulated in all three metastatic cell lines (Fig 3). The osteoblastic LM5 and LM8 cells showed the highest overlap in up-regulated genes (47%), reflected by five additional genes (FZD7, GLI2, NKD2, WNT1 and WNT11) that were found up-regulated in both cell lines. In summary, by compensation for the gene shift during culture, the analysis applied in the present study allowed the identification of genes involved in Hedgehog and WNT signaling pathways that were found up-regulated as a consequence of increased metastatic activity in all three cell line systems despite the limited stability of the investigated cell lines in culture.

## Discussion

The research in the field of OS is hampered by many circumstances. OS is a rare disease, morphologically heterogeneous and characterized by chromosomal instability that can include a chromothripsis-like pattern (CTLP) [3], which further increases the cell diversity within a given tumor specimen. There are a few established OS cell lines available that have been propagated *in vitro* for many doublings unknown in number since the isolation from the primary tumor. Therefore, there is an inherent risk for selection of certain cellular sub-clones by the *in vitro* propagation, e.g. through changes in cellular adhesion properties or proliferation rates as a consequence of ongoing genomic instability with the potential for altered gene expression and, as a result cellular *in vitro* and *in vivo* behavior. We therefore set out to investigate the gene expression stability during culture of three frequently used OS cell line systems, consisting of parental cell lines with low or moderate metastatic potential and derivatives thereof with increased metastatic potential. Two of the metastatic cell lines, the human LM5 [5] and mouse LM8 cells [9] were obtained by *in vivo* selection of parental SAOS and Dunn cells, respectively, whereas the 143B cells were derived from the HOS cells by Ki-ras transformation [30].

When we analyzed under high stringency the gene expression of the cell lines in early and late passages by microarray analysis, we realized that all cell lines, except the 143B cells, showed instability in gene expression. Interestingly, the *in vivo* selected LM5 and LM8 cells showed at high stringency (>2-fold; fdr<0.01) a 9- and 5-fold higher instability during serial passaging than the parental cell lines, whereas the Ki-ras transformed 143B cells were apparently more stable than the parental HOS cells. So far we have no explanation for these findings, and we can only speculate that *in vivo* selected highly metastatic cell lines were the result of higher genomic instability that might contribute to altered gene expression and consequently an increase in metastatic activity. This would be in line with the observed inverse correlation of genome complexity with patients’ survival in sarcomas and some other cancer entities [31,32]. Ki-ras transformed 143B cells, on the other hand, might have been *in vitro* selected during culture as a genetically relatively stable sub-clone characterized by higher proliferation rate (not shown) than the parental HOS cells.

Chromosomal instability resulting in altered gene expression during culture was also evident especially in the SAOS/LM5 cell system, where differentially expressed genes significantly clustered on certain chromosomes or even in cytobands. We therefore investigated chromosomal imbalance by assessing CN gains and losses by aCGH analysis in these cells during culture. Both cell lines showed indeed ongoing chromosomal imbalance that correlated in part with altered gene expression. Interestingly, a partial common change in both, the gene expression and the chromosomal imbalance, was observed during culture, indicating that these processes may not randomly occur in these two cell lines. Significant clustering of differentially expressed genes on distinct chromosomes (>2-fold; fdr<0.01) was less evident in the other two cell line systems, suggesting that other mechanisms are employed. Interestingly, genes encoding products important for "Focal adhesion" were deregulated in all cell lines, except for the apparently stable cell line 143B. Other pathways, e.g. "ECM–receptor interaction" were also significantly deregulated in some cell lines, indicating that indeed adhesive selection during culture on plastic ware may alter the composition of cell populations with time during *in vitro* culture. The high degree of chromosomal instability during *in vitro* culture observed here differs from observations in mouse xenograft sarcoma cultures also including OS samples [33].

In light of the observed instability in gene expression in OS cell lines during culture, the question arises whether a prediction of metastasis-relevant genes can be made by comparing differentially expressed genes in parental cell lines and their metastatic derivatives. In an analysis in which data sets of early and late passaged cells were pooled before they were analyzed for potential metastasis relevant genes, the number of genes regulated during selection for increased metastatic activity was 3-fold higher than that observed after serial passaging of the more unstable metastatic cell line. A pathway analysis of these pooled data sets revealed enrichment of genes involved in "Pathways in cancer" and "Wnt signaling pathway" in all three cell line systems, and in "Hedgehog signaling pathway" in two cell line systems. However, we also observed deregulation of genes belonging to these pathways during serial passaging of both the parental and metastatic cell lines. In an attempt to compensate for this shift in gene expression after serial passaging, we performed the comparison between high and low metastatic cell lines with all combinations of passaging data set comparisons (late/late, late/early, early/late and early/early). Resulting gene list for genes involved in "Pathways in cancer" revealed 14, 58 and 29 genes that were consistently (in all four comparisons) regulated in metastatic LM5, 143B and LM8 cells, respectively, compared to parental cell lines (Table H in S1 File). For the analysis of genes involved in "Hedgehog signaling" and "Wnt signaling pathway" the stringency was released to genes that were consistently regulated in at least three comparisons, as we observed deregulation of many of these genes also after serial passaging, especially in the SAOS/LM5 system. Here, we observed enrichment of 17, 24 and 17 up-regulated genes belonging to the "Hedgehog signaling" and/or "Wnt signaling pathway" in metastatic LM5, 143B and LM8 cells, respectively (Table K in S1 File and Fig 3).

The hedgehog (HH) signaling pathway includes among other components the secreted intercellular HH signaling proteins (IHH, DHH, SHH), the transmembrane HH receptors PTCH1 and -2 that, in the absence of ligands, constitutively repress the transmembrane receptor smoothened (SMO) and downstream the nuclear translocation of GLI family of zinc finger transcriptional regulators (for review see [34]; for KEGG pathway maps and gene lists see Fig C in S1 File). The canonical WNT signaling pathway includes among others the secreted WNT family of ligands, the frizzled (FZD) family of transmembrane receptors and co-receptors LRP5 and -6, the cytosolic DVL family of adaptor proteins that, in the presence of WNT ligand, releases GSK3 from the β-catenin destruction complex, which results in its nuclear translocation and the formation of a transcriptional complex together with TCF/LEF and finally activates target genes (for review see [35]). Both the HH and WNT signaling pathways play an important role in endochondral ossification and osteoblast maturation [29]. In OS there is accumulating evidence for the involvement of both pathways in tumor development and/or progression [36–39]. Here, we have identified common up-regulation of IHH, WNT10B and TCF7 in the metastatic LM5, LM8 and 143B cells compared to the parental cell lines.

In the HH signaling pathway, downstream of up-regulated IHH, PTCH1 or -2 were found up-regulated in LM5 and LM8 cells, respectively, together with GLI2. To this end, HH proteins have been shown to be over-expressed in OS cell lines compared to normal human bone [40] and PTCH1 was over-expressed in human OS biopsies and cell lines compared to normal human bone [40–42]. This indicates that an autocrine HH signaling may be involved in OS tumorigenesis. Downstream, GLI1 and -2 were shown over-expressed in OS cell lines compared to normal human bone [40–42] and GLI2 was also over-expressed in human OS biopsies [40,41]. Silencing of GLI2 inhibited OS tumor formation in nude mice [40,41] and arsenic trioxide, an inhibitor of GLI2 transcription, also reduced primary OS tumor formation in an OS mouse model [43]. Moreover, increased GLI2 expression correlated with poor survival of OS patients, indicating that GLI2 also contributes to tumor progression [42]. BMP2, a downstream target of GLI, was found up-regulated in 143B cells, and BMP2 was also increased in metastatic K7M2 compared to parental K12 OS cells [44]. Reanalysis of our Affymetrix arrays [4] revealed also increased PTCH1 expression in metastatic M132 and MG63-M8 compared to parental HUO9 and MG63 OS cells, respectively. Increased BMP2 expression was confirmed in K7M2 and also found in M132 OS cells. Taken together, the results point to an important role of up-regulated HH signaling in OS progression.

In the WNT signaling pathway, besides the commonly up-regulated WNT10B, several other WNT ligands were found to be up-regulated together with the FZD7 or -8 receptors (Fig 3). To this end, OS cells were shown to express several components of the WNT signaling pathway and the chemotactic response to exogenous WNT was increased in a mouse metastatic OS cell line compared to the parental cells with low metastatic potential, and increased WNT10B expression showed a trend (n = 44; P = 0.13) to shorter survival in patients [45,46]. An antibody to WNT1 induced cell death in sarcoma cells including OS [47]. Silencing of FHL2, a β-catenin interacting protein [48], reduced WNT signaling in OS cells, the expression of WNT5A and WNT10B and tumor formation and lung metastasis in a mouse OS model [49]. Silencing of WNT5A reduced the invasive properties of SAOS cells and WNT5A expression correlated with Enneking surgical stage and metastasis [50,51]. WNT5A was also over-expressed in metastatic sub-lines of the low metastatic human HUO9 OS cells [52] and in metastatic K7M2 compared to parental K12 cells, as revealed by the analysis of microarray data described in [4]. To our knowledge, no reports link altered FZD expression to OS tumor progression, but the expression of LRP5, a co-receptor for FZD, correlated with OS metastasis and high expression showed a trend (N = 44; P = 0.07) to shorter event free survival [46]. Interestingly, FZD7 was also found up-regulated in metastatic M132 and K7M2 compared to respective parental HUO9 and K12 OS cells [4]. Moreover, a dominant negative LRP5 mutant reduced tumor and metastasis formation in an experimental OS mouse model [53]. Downstream of the WNT receptors, the adaptor proteins DVL1 and DVL2 were up-regulated in 143B and LM8, respectively, and further downstream TCF7 was up-regulated in all 3 metastatic cell lines, and in metastatic M132 and K7M2 OS cells described in [4]. To this end, inhibition of TCF transcriptional activity reduced tumor growth and potentiated the effect of doxorubicin in an OS mouse model [54]. Taken together, our results imply that also up-regulated WNT signaling might contribute to metastasis formation in OS.

In conclusion, tumor derived cell lines are essential models to study biological aspects of malignancies, especially since they are amenable to genetic and pharmacological manipulation and an analysis of induced changes (e.g. DNA, gene expression, cell viability and morphology). However, cell lines inherently are the product of biological selection processes related to malignant transformation and/or immortality, but also of selection for and adaptation to *in vitro* conditions. The latter processes may be supported through an inherent genomic plasticity and promote the deviation of the cell line’s genotype and phenotype from these of the original tumor. Here, we have indeed observed instability of OS tumor cells during culture, which hampers the analysis of e.g. metastasis-relevant genes. However, by analyzing gene expression patterns of cells of different passages, we were able to identify up-regulated genes belonging to the "Hedgehog signaling pathway" and "Wnt signaling pathway" that appear to contribute to malignant transformation of OS cells.

In view of the here observed *in vitro* instability of OS cell lines, we would recommend that, in the future, the establishment of OS derived cells should not be based solely on *in vitro* propagation. Alternatively, freezing of viable stocks of biopsy or resection derived material should be envisaged. This can later be used for *in vivo* propagation in mice as patient-derived xenografts that have been shown to be genetically more stable [33].

## Supporting Information

S1 File Supporting tables and figures. **Table A in S1 File.** Culture conditions for cell lines. Difference (Δ) in passage number between late and early passage cells. Doublings were calculated based on 4.32 and 7.64 doublings at splitting ratios of 1:20 and 1:200, respectively. Table B in S1 File. Distribution of genes on chromosomes in the human and mouse arrays. n.p., not present in mouse; %, per cent of sum. Table C in S1 File. Primers used for real-time PCR analysis. Table D in S1 File. Chromosomal localization of differentially expressed genes after serial passaging and after selection for increased metastatic activity. Imbalances (p<0.01) of up- or down-regulated genes (>2fold; p<0.05) on chromosomes are listed when the sum of up- and down-regulated genes (in parenthesis) was >20. Analysis for metastasis-related genes was performed on pooled data sets obtained from early and late passaged cells. n; number of affected chromosomes. Preferential up-regulation is indicated in bold. Table E in S1 File. Chromosomal macro-aberrations after serial *in vitro* passaging of SAOS and LM5 cells. Commonly affected regions in SAOS and LM5 are indicated in yellow. Table F in S1 File. Correlation of regulated genes after serial *in vitro* passaging of SAOS and LM5 cells in microarray analysis with CN gains and losses in aCGH analysis. Fig A in S1 File. Chromosomal localization of regulated genes that correlate with CN gains and losses. A) Up-regulated genes in late compared to early SAOS that correlate with CN gain. B) Down-regulated genes in late compared to early SAOS that correlate with CN loss. C) Up-regulated genes in late compared to early LM5 that correlate with CN gain. D) Down-regulated genes in late compared to early LM5 that correlate with CN loss. Table G in S1 File. Chromosomal macro-aberrations after selection for increased metastatic activity in SAOS/LM5 cell system. The most affected regions are indicated in yellow. Fig B in S1 File. Real-time PCR analysis. Comparison of commonly up-regulated genes belonging to "Focal adhesion" in late versus early passaged SAOS and LM5 cells by microarray (MA) and real-time (qPCR) analyses. Table H in S1 File. Regulated (>2-fold; p<0.05) genes after selection for increased metastatic activity that belong to "Pathways in cancer" and are regulated under all passage-related comparisons. Table I in S1 File. Regulated genes (>2-fold; p<0.05) after serial *in vitro* passaging that belong to the "Hedgehog signaling pathway" and/or "WNT signaling pathway". Comparison is late versus early passage. Genes indicated in red are also regulated after selection for increased metastatic activity (Table K in S1 File). Table J in S1 File. Enrichment of regulated genes after selection for increased metastatic potential that belong to "Hedgehog signaling pathway" and "WNT signaling pathway" depending on individual comparisons of early and late passage data sets. Metastatic/parental; HH, "Hedgehog signaling pathway"; WNT, "WNT signaling pathway"; Total, total number of regulated (>2-fold; p<0.05) genes; Combined, combined gene lists of the four individual comparisons. Number of regulated genes (p value). Significant enrichment (p<0.05) is indicated in red. Table K in S1 File. Up-regulation of genes that belong to "Hedgehog signaling pathway" and/or "WNT signaling pathway" after selection for increased metastatic activity that are regulated (>2-fold; p<0.05) under at least three passage-related comparisons. Numbers in red indicate fold changes that do not meet the criteria >2-fold and/or p<0.05. Genes indicated in red were also regulated after serial *in vitro* passaging in either the parental or metastatic cell line (Table H). The means are calculated for all four comparisons. Fig C in S1 File. KEGG pathways.
(PDF)
