# Supplementary material for: Genomic Instability of Osteosarcoma Cell Lines in Culture: Impact on the Prediction of Metastasis Relevant Genes
Source: PLoS One. 2015 May 19;10(5):e0125611. doi: 10.1371/journal.pone.0125611 (PMC4438062; doi:10.1371/journal.pone.0125611)
Supplement: S1 File — Table A in S1 File. Culture conditions for cell lines. Difference (Δ) in passage number between late and early passage cells. Doublings were calculated based on 4.32 and 7.64 doublings at splitting ratios of 1:20 and 1:200, respectively. Table B in S1 File. Distribution of genes on chromosomes in the human and mouse arrays. n.p., not present in mouse; %, per cent of sum. Table C in S1 File. Primers used for real-time PCR analysis. Table D in S1 File. Chromosomal localization of differentially expressed genes after serial passaging and after selection for increased metastatic activity. Imbalances (p<0.01) of up- or down-regulated genes (>2fold; p<0.05) on chromosomes are listed when the sum of up- and down-regulated genes (in parenthesis) was >20. Analysis for metastasis-related genes was performed on pooled data sets obtained from early and late passaged cells. n; number of affected chromosomes. Preferential up-regulation is indicated in bold. Table E in S1 File. Chromosomal macro-aberrations after serial in vitro passaging of SAOS and LM5 cells. Commonly affected regions in SAOS and LM5 are indicated in yellow. Table F in S1 File. Correlation of regulated genes after serial in vitro passaging of SAOS and LM5 cells in microarray analysis with CN gains and losses in aCGH analysis. Fig A in S1 File. Chromosomal localization of regulated genes that correlate with CN gains and losses. A) Up-regulated genes in late compared to early SAOS that correlate with CN gain. B) Down-regulated genes in late compared to early SAOS that correlate with CN loss. C) Up-regulated genes in late compared to early LM5 that correlate with CN gain. D) Down-regulated genes in late compared to early LM5 that correlate with CN loss. Table G in S1 File. Chromosomal macro-aberrations after selection for increased metastatic activity in SAOS/LM5 cell system. The most affected regions are indicated in yellow. Fig B in S1 File. Real-time PCR analysis. Comparison of commonly up-regulated genes belong [file pone.0125611.s001.pdf]

Table A

Cell line	Passage (Δ)	Splitting	Doublings
human			
SAOS	38	1:20	164
LM5	36	1:20	156
HOS	41	1:200	314
143B	36	1:200	275
mouse			
Dunn	61	1:200	467
LM8	67	1:200	512

Table B

	Human		Mouse	
**Chr.**	**n**	**p(%)**	**n**	**p(%)**
1	2163	10.1	1331	5.8
2	1360	6.4	1891	8.2
3	1164	5.5	1101	4.8
4	819	3.8	1318	5.7
5	926	4.3	1340	5.8
6	1103	5.2	1247	5.4
7	1025	4.8	2004	8.7
8	774	3.6	1153	5
9	854	4	1339	4.9
10	837	3.9	1086	4.6
11	1353	6.3	1702	7.4
12	1093	5.1	771	3.3
13	382	1.8	916	4
14	668	3.1	868	3.8
15	669	3.1	878	3.8
16	883	4.1	746	3.3
17	1233	5.8	1118	4.8
18	321	1.5	571	2.5
19	1445	6.8	777	3.4
20	607	2.8	n.p.	n.p.
21	280	1.3	n.p.	n.p.
22	504	2.4	n.p.	n.p.
X	804	3.8	891	3.9
Y	72	0.3	26	0.1
**Sum**	**21339**		**23074**	
**Average**		**4.2**		**4.7**

Table C

Cyclin D1 (CCND1)
Forward primer: 5´- CCGTCCATGCGGAAGATC -3´
Reverse primer: 5´- ATGGCCAGCGGGAAGAC -3´

Integrin α_4 (ITGA4)
Forward primer: 5´- TTCGGAGCCAGCATACTACC -3´
Reverse primer: 5´- GCAGAATCAGACCGAAAAGC -3´

Parvin gamma (PARVG)
Forward primer: 5´- CTACCTCACTCCCAACTCTCCT -3´
Reverse primer: 5´- CTCAGTGTGCTCTTGGCATCCT -3´

Myosin regulatory light chain 10 (MYL10)
Forward primer: 5´- GGATTACAGGCACCGAGAAGAG -3´
Reverse primer: 5´- TGAGCCGAGATCATGCCATTGC -3´

Glyceraldehyde-3-phosphate dehydrogenase (GAPDH)
Forward primer: 5´-AGGGCTGCTTTTAACTCTGGT-3′
Reverse primer: 5′-CCCCACTTGATTTTGGAGGGA-3′

Table D

Serial *in vitro* passaging		
**(up/down/total)**	**n (up/down)**	**Affected chromosomes (up + down)**
SAOS (810/487/1297)	4 (2/2)	**1 (145)**; 5 (53); 6 (68); **12 (83)**
LM5 (1035/912/1947)	7 (3/4)	**4 (121)**; 9 (101); 10 (113); **12 (160)**; 14 (66); 18 (33); **21 (46)**
HOS (705/400/1105)	2 (0/2)	17 (49); 19 (79)
143B (338/92/430)	0	
Dunn (313/313/626)	3 (2/1)	9 (36); **14 (28); 18 (23)**
LM8 (327/338/665)	1 (1/0)	**X (58)**
**Metastatic comparison**		
LM5/SAOS (1283/1295/2578)	12 (6/6)	**1 (313); 2 (181); 4 (130); 5 (142); 6 (130); 7 (125)**; 8 (121); 10 (95); 16 (83); 19 (150); 21 (36); X (119)
143B/HOS (1833/1967/3800)	9 (5/4)	**1 (419); 3 (205)**; 6 (197); **10 (152)**; 13 (65); 15 (116); 17 (216); **18 (81); 20 (120)**
LM8/Dunn (1363/660/2029)	6 (4/2)	**4 (147)**; 6 (141); 9 (128); **12 (107); 16 (89); 18 (84)**

Table E

	SAOS					LM5		
	early	late	late/early			early	late	late/early
1q23.2-q42.13	normal	gain	gain					
					2p25.2-p25.1	normal	gain	gain
2q11.2	gain	normal	loss					
2q13	normal	loss	loss					
					2q37.3	gain	normal	loss
3q28-q29	gain	normal	loss		3q28	gain	normal	loss
					4p16.3-p16.1	normal	gain	gain
4q13.1-q13.3	gain	normal	loss					
					5p15.33	normal	gain	gain
					5p14.3	normal	gain	gain
					5p14.1	normal	gain	gain
6p21.32-p21.1	gain	normal	loss					
					7p21.1-p11.1	gain	normal	loss
7q36.1-q36.3	gain	normal	loss					
9p12-p11.1	normal	gain	gain					
9q21.13	gain	normal	loss		9q21.13	gain	normal	loss
9q21.33-q32	gain	normal	loss		9q21.31-q31.3	gain	normal	loss
					9q34.2-q34.3	gain	normal	loss
					10p	normal	loss	loss
12p13.33	loss	normal	gain		12p13.33	loss	normal	gain
12p13.32-p11.1	normal	gain	gain		12p13.32-p11.1	normal	gain	gain
14q22.3-q32.33	gain	normal	loss		14q22.3-q32.2	gain	normal	loss
18q23	normal	loss	loss					
19q13.11-q13.12	normal	gain	gain					
					21q	normal	gain	gain
Xp22.33-p21.2	gain	normal	loss		Xp22.33-p22.31	normal	gain	gain
Xq11.2-q27.1	gain	normal	loss					

Table F

	delta CNA bases (directional)	genome fraction	top genes	genes expected	genes observed	sum expected	sum observed	cumulative CNA gene ratio
**SAOS up**	137298826	0.044	1111	49	116			
**SAOS down**	237707052	0.077	686	53	86	102	202	2.0
**LM5 up**	122213823	0.039	1489	59	175			
**LM5 down**	212629685	0.069	1277	88	154	146	329	2.2
**HG19 bases**	3095676202							

Figure 1

A)

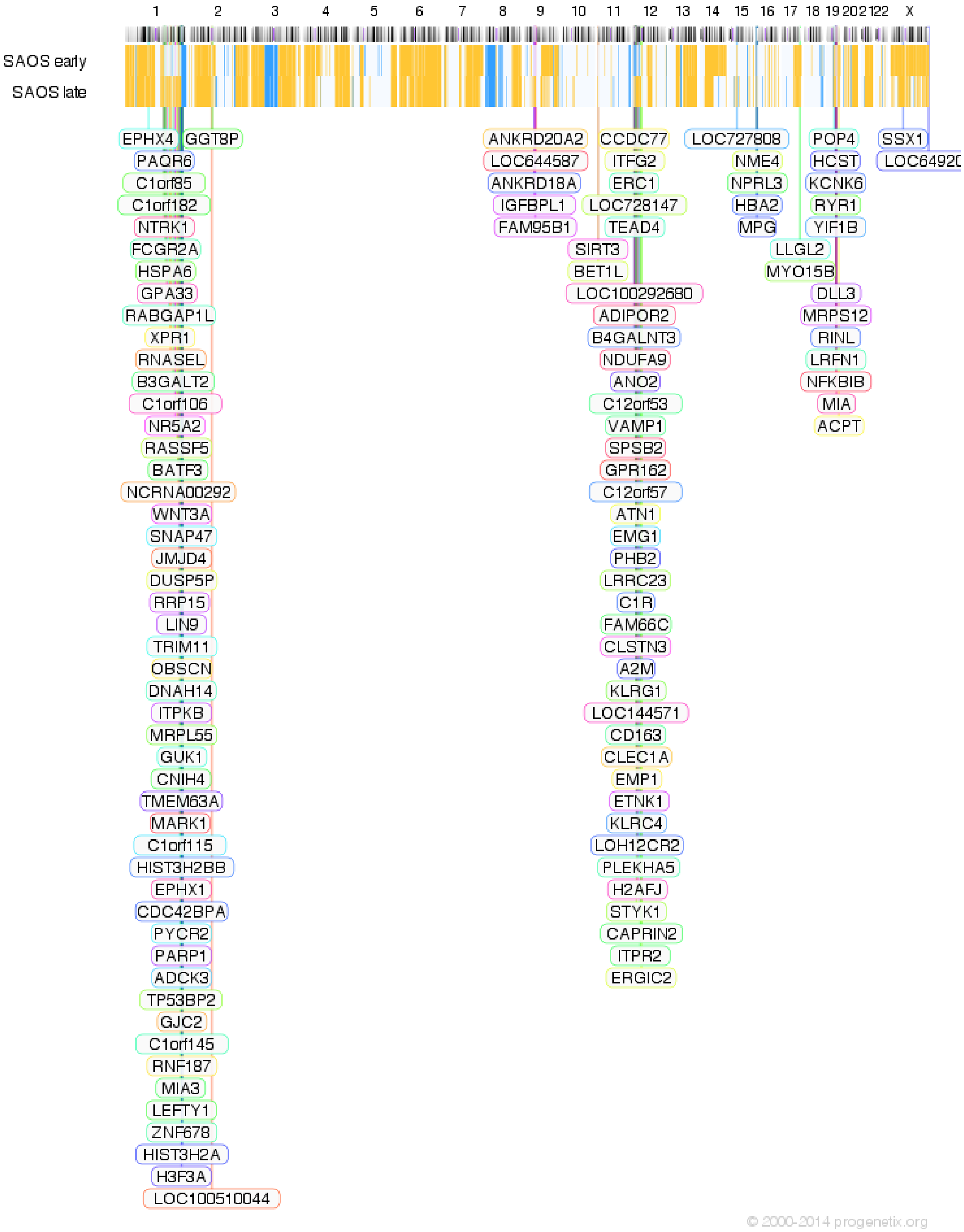
1 2 3 4 5 6 7 8 9 10 11 12 13 14 15 16 17 18 19 20 21 22 X
SAOS early
SAOS late
EPHX4
GGT8P
PAQR6
C1orf85
C1orf182
NTRK1
FCGR2A
HSPA6
GPA33
RABGAP1L
XPR1
RNASEL
B3GALT2
C1orf106
NR5A2
RASSF5
BATF3
NCRNA00292
WNT3A
SNAP47
JMJD4
DUSP5P
RRP15
LIN9
TRIM11
OBSCN
DNAH14
ITPKB
MRPL55
GUK1
CNIH4
TMEM63A
MARK1
C1orf115
HIST3H2BB
EPHX1
CDC42BPA
PYCR2
PARP1
ADCK3
TP53BP2
GJC2
C1orf145
RNF187
MIA3
LEFTY1
ZNF678
HIST3H2A
H3F3A
LOC100510044
ANKRD20A2
LOC644587
ANKRD18A
IGFBPL1
FAM95B1
CCDC77
ITFG2
ERC1
LOC728147
TEAD4
SIRT3
BET1L
LOC100292680
ADIPOR2
B4GALNT3
NDUFA9
ANO2
C12orf53
VAMP1
SPSB2
GPR162
C12orf57
ATN1
EMG1
PHB2
LRRC23
C1R
FAM66C
CLSTN3
A2M
KLRG1
LOC144571
CD163
CLEC1A
EMP1
ETNK1
KLRC4
LOH12CR2
PLEKHA5
H2AFJ
STYK1
CAPRIN2
ITPR2
ERGIC2
LOC727808
NME4
NPRL3
HBA2
MPG
LLGL2
MYO15B
POP4
HCST
KCNK6
RYR1
YIF1B
DLL3
MRPS12
RINL
LRFN1
NFKBIB
MIA
ACPT
SSX1
LOC6492
© 2000-2014 progenetix.org

B)

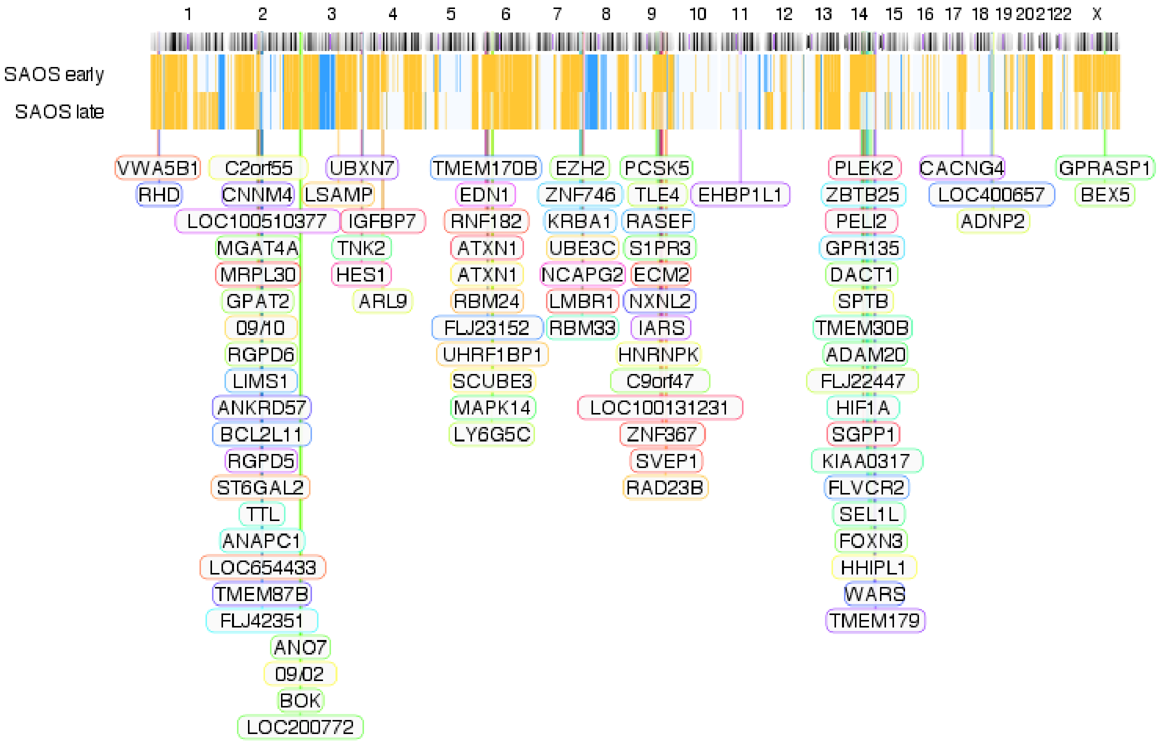

© 2000-2014 progenetix.org

C)

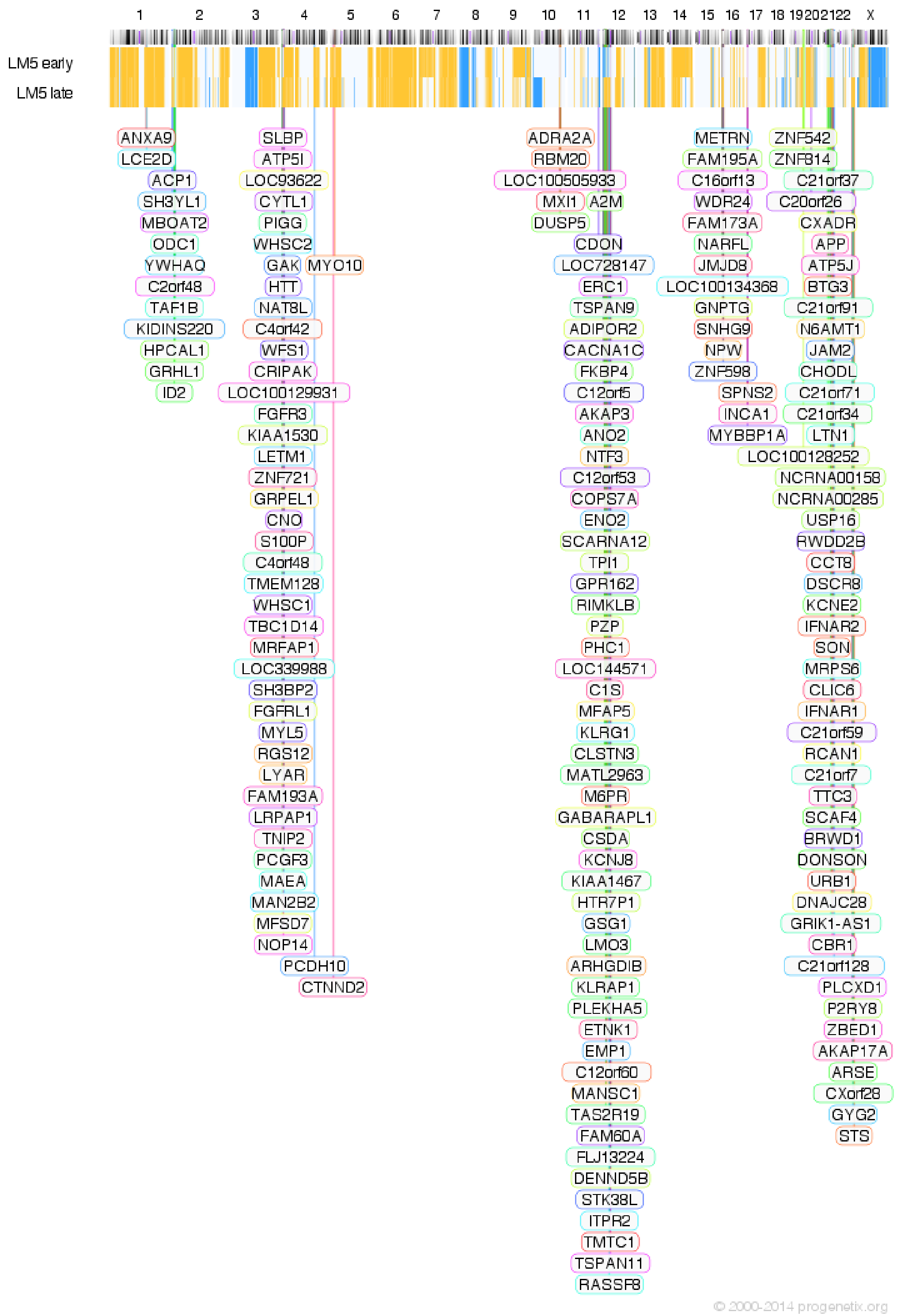
1 2 3 4 5 6 7 8 9 10 11 12 13 14 15 16 17 18 19 20 21 22 X
LM5 early
LM5 late
ANXA9
LCE2D
ACP1
SH3YL1
MBOAT2
ODC1
YWHAQ
C2orf48
TAF1B
KIDINS220
HPCAL1
GRHL1
ID2
SLBP
ATP5I
LOC93622
CYTL1
PIGG
WHSC2
GAK
MYO10
HTT
NAT8L
C4orf42
WFS1
CRIPAK
LOC100129931
FGFR3
KIAA1530
LETM1
ZNF721
GRPEL1
CNO
S100P
C4orf48
TMEM128
WHSC1
TBC1D14
MRFAP1
LOC339988
SH3BP2
FGFRL1
MYL5
RGS12
LYAR
FAM193A
LRPAP1
TNIP2
PCGF3
MAEA
MAN2B2
MFSD7
NOP14
PCDH10
CTNND2
ADRA2A
RBM20
LOC100505933
MXI1
A2M
DUSP5
CDON
LOC728147
ERC1
TSPAN9
ADIPOR2
CACNA1C
FKBP4
C12orf5
AKAP3
ANO2
NTF3
C12orf53
COPS7A
ENO2
SCARNA12
TPI1
GPR162
RIMKLB
PZP
PHC1
LOC144571
C1S
MFAP5
KLRG1
CLSTN3
MATL2963
M6PR
GABARAPL1
CSDA
KCNJ8
KIAA1467
HTR7P1
GSG1
LMO3
ARHGDIB
KLRAP1
PLEKHA5
ETNK1
EMP1
C12orf60
MANSC1
TAS2R19
FAM60A
FLJ13224
DENND5B
STK38L
ITPR2
TMTC1
TSPAN11
RASSF8
METRN
FAM195A
C16orf13
WDR24
FAM173A
NARFL
JMJD8
LOC100134368
GNPTG
SNHG9
NPW
ZNF598
SPNS2
INCA1
MYBBP1A
ZNF542
ZNF814
C21orf37
C20orf26
CXADR
APP
ATP5J
BTG3
C21orf91
N6AMT1
JAM2
CHODL
C21orf71
C21orf34
LTN1
LOC100128252
NCRNA00158
NCRNA00285
USP16
RWDD2B
CCT8
DSCR8
KCNE2
IFNAR2
SON
MRPS6
CLIC6
IFNAR1
C21orf59
RCAN1
C21orf7
TTC3
SCAF4
BRWD1
DONSON
URB1
DNAJC28
GRIK1-AS1
CBR1
C21orf128
PLCXD1
P2RY8
ZBED1
AKAP17A
ARSE
CXorf28
GYG2
STS
© 2000-2014 progenetix.org

D)

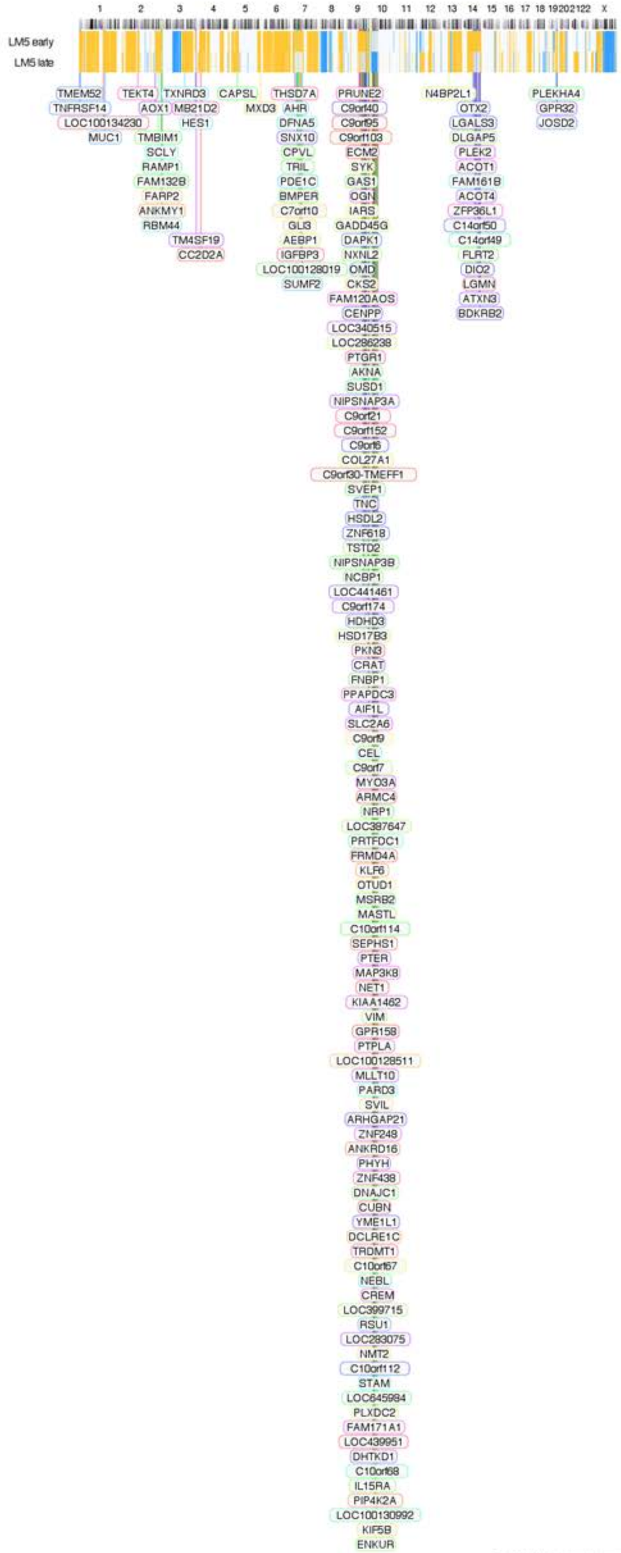

© 2000-2014 progenetix.org

Table G

	SAOS	LM5	LM5/SAOS
1q23.1-q23.2	normal	gain	gain
1q31.1-q31.3	normal	gain	gain
1q42.2-q44	loss	normal	gain
2q32.2-q33.1	gain	normal	loss
3p26.3-p14.3	gain	normal	loss
4q32.1-q34.3	gain	normal	loss
5p15.32-p13.3	normal	gain	gain
8q23.2-q23.3	gain	normal	loss
8q24.23-q24.3	gain	normal	loss
15q23-q24.1	normal	gain	gain
18p11.32	normal	loss	loss
18p11.31	normal	gain	gain
20p13-p12.3	normal	gain	gain
20p12.1	normal	gain	gain
20q13.12	gain	loss	loss
20q13.13-q13.33	gain	normal	loss
Xq	normal/gain	loss/normal	loss

Figure 2

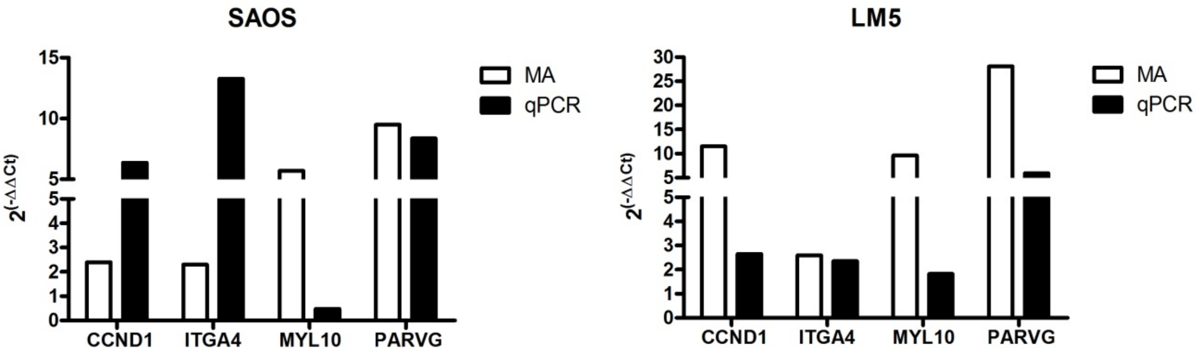
SAOS
15
10
5
5
4
3
2
1
0
$2^{(-\Delta\Delta Ct)}$
CCND1
ITGA4
MYL10
PARVG
MA
qPCR
LM5
30
25
20
15
10
5
5
4
3
2
1
0
$2^{(-\Delta\Delta Ct)}$
CCND1
ITGA4
MYL10
PARVG
MA
qPCR

Table H

			LM5/SAOS				
			L/L	L/E	E/L	E/E	Mean
Gene	KEGG	**Up-regulated in LM5**					
WNT1	7471	wingless-type MMTV integration site family, member 1	7	11.3	10.9	17.5	11.7
FIGF	2277	c-fos induced growth factor (vascular endothelial growth factor D)	4.9	8.9	7.8	14.1	8.9
PTCH1	5727	patched 1	9.5	11.5	5.3	3.8	7.5
RASSF5	83593	Ras association (RalGDS/AF-6) domain family member 5	6.4	14.3	2.2	4.9	7.0
FGF5	2250	fibroblast growth factor 5	5.5	3.7	6.9	4.8	5.2
FOXO1	2308	forkhead box O1	3	3.2	2.3	2.5	2.8
FH	2271	fumarate hydratase	2.2	2.6	2.3	2.7	2.5
		**Down-regulated in LM5**					
TCF7L1	83439	transcription factor 7-like 1 (T-cell specific, HMG-box)	100	100	100	100	100.0
PLCG2	5336	phospholipase C, gamma 2 (phosphatidylinositol-specific)	33.3	6.3	33.3	5.3	19.6
PPARG	5468	peroxisome proliferator-activated receptor gamma	11.1	14.3	16.7	20	15.5
FGF1	2246	fibroblast growth factor 1 (acidic)	2.1	4	20	33.3	14.9
KIT	3815	v-kit Hardy-Zuckerman 4 feline sarcoma viral oncogene homolog	4.3	8.3	2.8	4.3	4.9
FZD8	8325	frizzled family receptor 8	4	5.6	3	4.2	4.2
STK4	6789	serine/threonine kinase 4	2.8	3.4	2.6	3.2	3.0

			LM8/Dunn				
			L/L	L/E	E/L	E/E	Mean
		**Up-regulated in LM8**					
EGFR	13649	epidermal growth factor receptor	25.9	19	75	57	44.2
DAPK2	13143	death-associated protein kinase 2	26.4	7.1	24	6.8	16.1
MMP2	17390	matrix metallopeptidase 2	10	7.2	13.5	10	10.2
WNT8B	22423	wingless related MMTV integration site 8b	14.1	8.1	12.1	5.9	10.1
BIRC3	11796	baculoviral IAP repeat-containing 3	6.7	10.3	7.3	10.5	8.7
PTCH2	19207	patched homolog 2	3.5	15.3	2.1	8.3	7.3
WNT1	22408	wingless-related MMTV integration site 1	8.5	6.1	7.7	5.5	7
MAPK1	26413	mitogen-activated protein kinase 1	2.3	7	2.4	7.3	4.8
CSF1R	12978	colony stimulating factor 1 receptor	5.1	5	4.4	3.9	4.6
GLI2	14633	GLI-Kruppel family member GLI2	3.8	4.4	4.3	4.8	4.3
PIAS1	56469	protein inhibitor of activated STAT 1	3.8	5	2.2	2.9	3.5
SUFU	24069	suppressor of fused homolog (Drosophila)	2.4	4.3	2.3	4.2	3.3
TRAF3	22031	TNF receptor-associated factor 3	3.1	3.5	2.6	3	3.1
FZD7	14369	frizzled homolog 7 (Drosophila)	3.2	3.2	2.5	2.1	2.8
		**Down-regulated in LM8**					
PDGFB	18591	platelet derived growth factor, B polypeptide	100	100	50	100	87.5
LAMA1	16772	laminin, alpha 1	111	50	9.1	5	43.8
VEGFC	22341	vascular endothelial growth factor C	33.3	100	5.3	10	37.2
PPARG	19016	peroxisome proliferator activated receptor gamma	50	10	50	10	30
LEF1	16842	lymphoid enhancer binding factor 1	10	33.3	50	20	28.3
KITL	17311	kit ligand	9.1	16.7	6.3	11.1	10.8
PLCG2	234779	phospholipase C, gamma 2	12.5	7.7	8.3	4.8	8.3
CSF2RA	12982	colony stimulating factor 2 receptor, alpha, low-affinity (granulocyte-	5	10	7.7	10	8.2
ITGA3	16400	integrin alpha 3	6.3	6.3	4.3	4.3	5.3
PRKCC	18752	protein kinase C, gamma	6.7	2.4	6.3	2.2	4.4
NFKB1	18033	nuclear factor of kappa light polypeptide gene enhancer in B-cells 1, p	4	3.8	2.7	2.6	3.3
STAT3	20848	signal transducer and activator of transcription 3	2.8	3.1	3.2	2.9	3
MITF	17342	microphthalmia-associated transcription factor	2.6	3.3	2.2	2.9	2.8
LAMA4	16775	laminin, alpha 4	2.9	3.2	2.1	2.4	2.7
MYC	17869	myelocytomatosis oncogene	2.5	2.8	2.1	2.2	2.4

			143B/HOS				
			L/L	L/E	E/L	E/E	Mean
**Up-regulated in 143B**							
MMP1	4312	matrix metallopeptidase 1 (interstitial collagenase)	1087	649	716	433	721.3
RAC2	5880	rho family, small GTP binding protein Rac2	105	58.7	98.5	54.5	79.2
HHIP	64399	hedgehog interacting protein	19.9	31.4	34.7	57.2	35.8
FGF5	2250	fibroblast growth factor 5	42.9	17.9	50.9	22.1	33.5
WNT7A	7476	wingless-type MMTV integration site family, member 7A	18.5	16.6	17.4	15.7	17.1
BMP2	650	bone morphogenetic protein 2	4.3	33	3.3	26.5	16.8
COL4A6	1288	collagen, type IV, alpha 6	17.2	19	10.1	11	14.3
IL8	3576	interleukin 8	17.9	14.7	6	5.1	10.9
LAMB3	3914	laminin, beta 3	12.1	8.4	10.7	7.3	9.6
LEF1	51176	lymphoid enhancer-binding factor 1	2.2	16.7	2.2	17.1	9.6
FZD8	8325	frizzled family receptor 8	9.8	8.5	8.5	7.3	8.5
WNT7B	7477	wingless-type MMTV integration site family, member 7B	2.7	15.4	2.3	12.6	8.3
ITGA2	3673	integrin, alpha 2 (CD49B, alpha 2 subunit of VLA-2 receptor)	10.5	10	5.4	5.5	7.9
PTGS2	5743	prostaglandin-endoperoxide synthase 2	7.6	7.7	4.9	5	6.3
FOS	2353	FBJ murine osteosarcoma viral oncogene homolog	6.4	6.9	4	4.2	5.4
FGF7	2252	fibroblast growth factor 7	3.7	7	3.4	6.7	5.2
TGFBR2	7048	transforming growth factor, beta receptor II (70/80kDa)	6.3	6.6	3.8	2.9	4.9
JUN	3725	jun proto-oncogene	4.5	4.6	4.1	4.2	4.4
BCL2	596	B-cell CLL/lymphoma 2	3	3.8	3.2	4.2	3.6
PIK3CG	5294	phosphoinositide-3-kinase, catalytic, gamma polypeptide	2.5	4.4	2.3	4	3.3
TCF7	6932	transcription factor 7 (T-cell specific, HMG-box)	2.2	4.3	2.4	3.1	3.0
NRAS	4893	neuroblastoma RAS viral (v-ras) oncogene homolog	2.3	2.1	3.6	3.1	2.8
WNT2B	7482	wingless-type MMTV integration site family, member 2B	2.9	2.9	2.4	2.4	2.7
CCNE2	9134	cyclin E2	2.3	2.4	2.6	2.7	2.5
MMP9	4318	matrix metallopeptidase 9	2.6	2.9	2.2	2.4	2.5
DVL1	1855	dishevelled, dsh homolog 1 (Drosophila)	2.2	2.5	2.1	2.1	2.2
**Down-regulated in 143B**							
PDGFRB	5159	platelet-derived growth factor receptor, beta polypeptide	333	333	333	250	312
EGF	1950	epidermal growth factor	50	50	20	25	36
NOS2	4843	nitric oxide synthase 2, inducible	33.3	20	33.3	25	27.9
GLI1	2735	GLI family zinc finger 1	25	3.3	50	5.6	21
RUNX1T1	862	runt-related transcription factor 1; translocated to, 1 (cyclin D-related)	25	12.5	25	12.5	19
FN1	2335	fibronectin 1	7.7	8.3	10	11.1	9.3
STAT5A	6776	signal transducer and activator of transcription 5A	8.3	11.1	6.7	9.1	8.8
JUP	3728	junction plakoglobin	11.1	6.7	10	5.9	8.4
FLT3	2322	fms-related tyrosine kinase 3	11.1	6.3	7.1	4.2	7.2
LAMC3	10319	laminin, gamma 3	7.7	7.7	5.9	5.6	6.7
FOXO1	2308	forkhead box O1	4.2	7.1	4	6.7	5.5
FGF18	8817	fibroblast growth factor 18	8.3	3.7	6.7	3	5.4
TGFB2	7042	transforming growth factor, beta 2	5.6	6.7	4.2	5	5.3
FGF1	2246	fibroblast growth factor 1 (acidic)	5	7.1	2.9	4	5
ARAF	369	v-raf murine sarcoma 3611 viral oncogene homolog	5	5.9	3.7	4.3	5
COL4A1	1282	collagen, type IV, alpha 1	4.2	5.9	4	5.6	4.9
TRAF5	7188	TNF receptor-associated factor 5	3.7	4.3	4.8	5.6	4.6
PDGFB	5155	platelet-derived growth factor beta polypeptide	3.6	6.3	2.9	5	4.4
BMP4	652	bone morphogenetic protein 4	2.9	6.7	2.3	5.3	4.3
COL4A4	1286	collagen, type IV, alpha 4	3.6	2.5	6.7	4.3	4.3
PTCH1	5727	patched 1	4.2	4.3	4	4.2	4.2
FZD2	2535	frizzled family receptor 2	4.2	4	3.2	3	3.6
LAMA5	3911	laminin, alpha 5	3.2	2.4	4.5	3.6	3.4
TCF7L1	83439	transcription factor 7-like 1 (T-cell specific, HMG-box)	2.9	3.1	3.3	3.6	3.2
WNT3	7473	wingless-type MMTV integration site family, member 3	3.6	2.4	3.8	2.6	3.1
IKBKB	3551	inhibitor of kappa light polypeptide gene enhancer in B-cells, kinase beta	3	2.5	2.7	2.3	3
ITGAV	3685	integrin, alpha V (vitronectin receptor, alpha polypeptide, antigen CD51)	2.4	2.3	3.2	3.1	2.8
ARNT	405	aryl hydrocarbon receptor nuclear translocator	2.4	3.3	2.1	3	2.7
COL4A2	1284	collagen, type IV, alpha 2	2.6	2.8	2.7	2.9	2.7
ERBB2	2064	v-erb-b2 erythroblastic leukemia viral oncogene homolog 2	2.5	2.2	3.1	2.8	2.7
CTNNA1	1495	catenin (cadherin-associated protein), alpha 1, 102kDa	2.6	2.6	2.3	2.4	2.5
LAMC1	3915	laminin, gamma 1 (formerly LAMB2)	2.2	2.6	2.3	2.7	2.4

Table I

SAOS_up	BMP2, BMP7, CCND1, DAAM2, DKK1, HHIP, IHH, NFATC1, WIF1, WNT3A, WNT6, WNT11
SAOS_down	NKD2, MAPK9, SFRP2
LM5_up	BMP6, CAMK2B, CCND1, DAAM2, GLI2, IHH, LRP5, PLCB1, PRKACG, PPP2R1B, SFRP1, WIF1, WNT6
LM5_down	BMP7, FZD7, GAS1, GLI3, LEF1, NFATC1, PRICKLE1, WNT2B
HOS_up	APC2, BMP2, BMP7, CCND2, CXXC4, FZD10, GLI1, LEF1, NFATC4, NKD2, PLCB1, WIF1, WNT3A, WNT4, WNT6, WNT7B, WNT10B, WNT11
HOS_down	
143B_up	APC, CXXC4, ROCK1, ROCK2
143B_down	
Dunn_up	CCND2, LEF1, PRICKLE1, RHOA, SFRP4, WIF1, WNT6, WNT10A
Dunn_down	
LM8_up	BMP7, CER1, MAPK10, SFRP4, TCF7L1, WNT11
LM8_down	DAAM2, FOSL1, FZD8, NFATC4, RAC3

Table J

	L/L	E/L	L/E	E/E	combined
			**LM5/SAOS**		
			**Up-regulation**		
Total	1613	1368	1848	1291	3174
HH	10 (1.8E-2)	6 (2.4E-1)	13 (5.1E-3)	10 (5.7E-3)	14 (6E-2)
WNT	16 (1.3E-1)	10 (5.5E-1)	17 (3.5E-1)	15 (6.9E-2)	23 (5.6E-1)
			**Down-regulation**		
Total	1685	1634	1462	1215	2942
HH	7 (3.4E-1)	5 (6.8E-1)	4 (7.9E-1)	2 (9.7E-1)	8 (7.2E-1)
WNT	17 (2.1E-1	21 (1.9E-2)	15 (2.1E-1)	17 (1.4E-2)	30 (6.3E-2)
			**LM8/Dunn**		
			**Up-regulation**		
Total	2008	1324	3025	2067	4174
HH	8 (2E-1)	8 (3E-2)	13 (4.6E-2)	16 (9.5E-5)	19 (4.6E-3)
WNT	23 (1.2E-2)	15 (4E-2)	28 (5.2E-2)	27 (1.4E-3)	44 (6E-4)
			**Down-regulation**		
Total	1376	944	3223	2021	4060
HH	4 (7.2E-1)	4 (4.4E-1)	7 (8.3E-1)	5 (7.5E-1)	11 (5.2E-1)
WNT	9 (8E-1)	6 (8.1E-1)	20 (8E-1)	8 (9.9E-1)	23 (8.8E-1)
			**143B/HOS**		
			**Up-regulation**		
Total	1725	1888	1863	1821	2914
HH	13 (2.1E-3)	13 (5.2E-3)	11 (2.2E-2)	9 (1.1E-1)	14 (3.1E-2)
WNT	21 (3E-2)	23 (2.6E-2)	24 (6E-3)	25 (2.8E-3	35 (3E-3)
			**Down-regulation**		
Total	1999	2341	1954	2035	3342
HH	10 (1.1E-1)	12 (4.2E-2)	7 (4.6E-1)	8 (3.2E-1)	12 (2.5E-1)
WNT	19 (2.8E-1)	28 (8.2E-3)	14 (7.2E-1)	15 (6.5E-1)	29 (1.8E-1)

Table K

Up-regulated in LM5						
	**L/L**	**E/L**	**L/E**	**E/E**	**Mean**	**Comp.**
WIF1	124.2	19.2	468.1	67.1	169.7	4
IHH	21.2	1	58.5	2.8	20.9	3
PLCB1	23.9	4	26.1	4.1	14.5	4
WNT1	7	10.9	11.3	17.5	11.7	4
BMP6	17.4	3.2	15.8	2.8	9.8	4
NKD2	8.5	4.4	13.7	7.1	8.4	4
PTCH1	9.5	5.3	11.5	3.8	7.5	4
WNT6	5.8	1	14	2.4	5.8	3
DAAM2	2.8	0.9	11.7	3.6	4.8	3
NKD1	2.9	2.1	5.7	4.1	3.7	4
HHIP	2	2.5	4.3	5.3	3.5	3
GLI2	3.3	2	5.4	3.2	3.5	3
WNT10B	3.2	1.8	5.3	2.9	3.3	3
FZD7	2.6	5.3	1.4	2.8	3.0	3
WNT11	2.1	1.3	4.3	2.6	2.6	3
WNT5A	4	2.4	2.4	1.4	2.6	3
TCF7	2.2	1.8	2.9	2.4	2.3	3

Up-regulated in 143B						
RAC2	105	98	59	55	79.3	4
HHIP	19.9	34.7	31.4	57	35.8	4
WNT7A	18.5	17.4	16.6	15.7	17.1	4
BMP2	4.3	3.3	33	26.5	16.8	4
LEF1	2.2	2.2	16.7	17.1	9.6	4
BMP6	6.8	6.2	13.1	12	9.5	4
BMP7	3.7	4.8	12.1	15	8.9	4
FZD8	9.8	8.5	8.5	7.3	8.5	4
WNT7B	2.7	2.3	15.4	12.6	8.3	4
DKK1	8.2	8.3	5.3	5.2	6.8	4
FOSL1	8	6.8	5.2	4.4	6.1	4
JUN	4.5	4.1	4.6	4.2	4.4	4
IHH	4.5	4.6	3.9	3.9	4.2	4
NKD1	2.7	3	4.2	4.8	3.7	4
TCF7	2.6	2.4	4.3	3.1	3.1	4
WNT10B	2.1	1.9	4.2	4	3.1	3
SOX17	2.1	2.2	3	3.3	2.7	4
WNT2B	2.9	2.4	2.9	2.4	2.7	4
PRKACB	3.1	2.3	3	2	2.6	3
SFRP1	2.6	1.9	3.1	2.2	2.5	3
CTNNBIP1	2.5	3	1.9	2.3	2.4	3
SUFU	2.1	2.1	2.7	2.3	2.3	3
DVL1	2.2	2.1	2.5	2.3	2.3	4
MYC	1.9	2.3	2.1	2.6	2.2	3

**Up-regulated in LM8**						
VANGL1	67.4	69.9	15.3	13.1	41.4	4
WNT8B	14.1	12.1	8.1	5.9	10.1	4
NFATC4	4.2	14.8	3.3	13.2	8.9	4
PPP2R5C	14.1	6	10	3.6	8.4	4
PTCH2	3.5	2.2	15.3	8.3	7.3	4
IHH	4.9	6.5	7.7	10	7.3	3
NKD2	11.9	12.2	2	2.2	7.1	3
WNT1	8.5	7.7	6.1	5.5	7.0	4
SFRP4	5.4	2.1	14.5	5.6	6.9	4
WNT11	4.5	2	11.8	4.5	5.7	3
GLI2	3.8	4.3	4.4	4.8	4.3	4
APC2	5.4	5.7	1.9	2.1	3.8	3
SUFU	2.4	2.3	4.3	4.2	3.3	4
DVL2	3.4	2.5	3.6	2.7	3.1	3
FZD7	3.2	2.5	3.2	2.1	2.8	4
WNT10B	2.7	2.2	2.8	2.3	2.5	3
TCF7	2.4	2.6	2	2.1	2.3	3

Figure 3

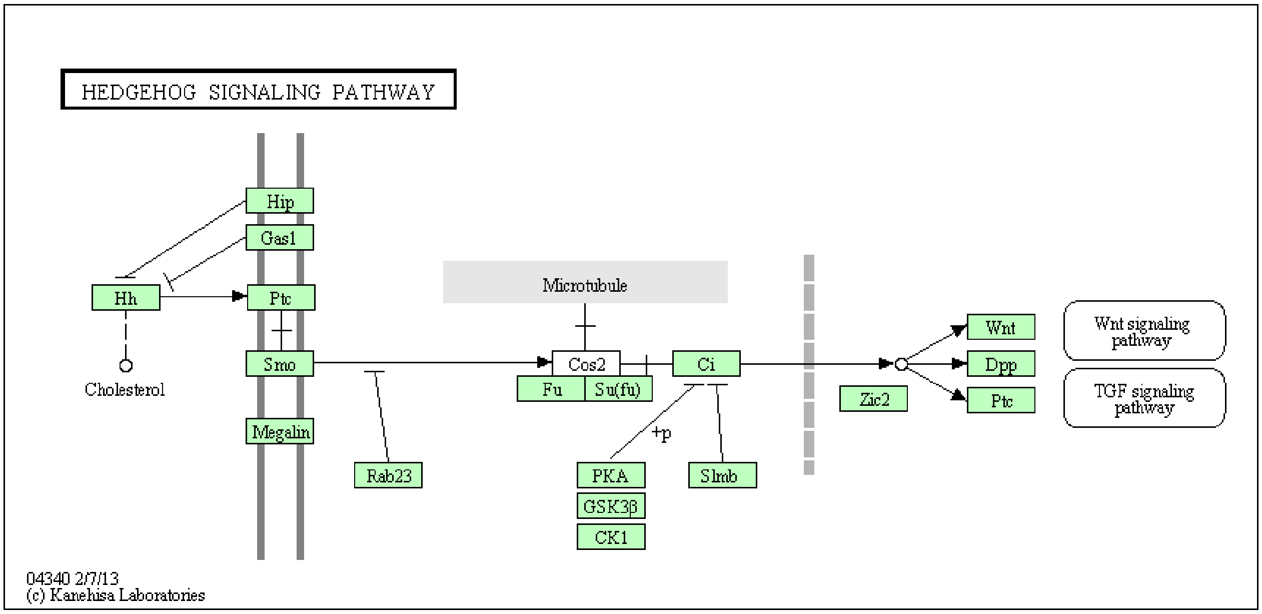

For gene list see: http://www.genome.jp/dbget-bin/www_bget?pathway+hsa04340

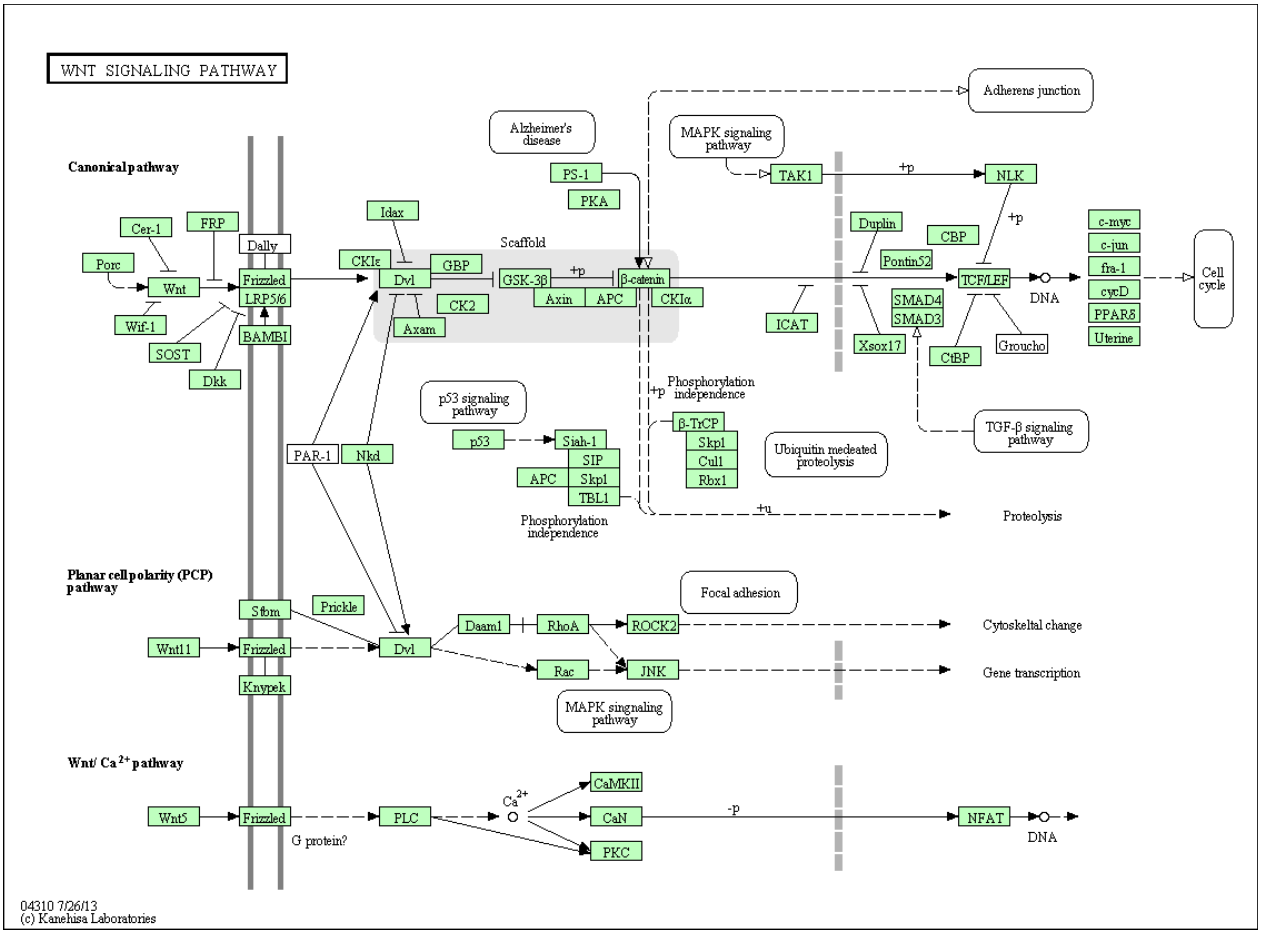

For gene list see: http://www.genome.jp/dbget-bin/www_bget?pathway+hsa04310
